# Highly active and durable methanol oxidation electrocatalyst based on the synergy of platinum–nickel hydroxide–graphene

**DOI:** 10.1038/ncomms10035

**Published:** 2015-11-25

**Authors:** Wenjing Huang, Hongtao Wang, Jigang Zhou, Jian Wang, Paul N. Duchesne, David Muir, Peng Zhang, Na Han, Feipeng Zhao, Min Zeng, Jun Zhong, Chuanhong Jin, Yanguang Li, Shuit-Tong Lee, Hongjie Dai

**Affiliations:** 1 Institute of Functional Nano and Soft Materials (FUNSOM), Soochow University, 199 Ren'ai Road, Suzhou Industrial Park, Suzhou 215123, China; 2 State Key Laboratory of Silicon Materials and School of Materials Science and Engineering, Zhejiang University, Hangzhou, Zhejiang 310027, China; 3 Canadian Light Source Inc., Saskatoon, Saskatchewan, Canada S7N 0X4; 4 Department of Chemistry, Dalhousie University, Halifax, Canada NS B3H 4R2; 5 Department of Chemistry, Stanford University, Stanford, California 94305, USA

## Abstract

Active and durable electrocatalysts for methanol oxidation reaction are of critical importance to the commercial viability of direct methanol fuel cell technology. Unfortunately, current methanol oxidation electrocatalysts fall far short of expectations and suffer from rapid activity degradation. Here we report platinum–nickel hydroxide–graphene ternary hybrids as a possible solution to this long-standing issue. The incorporation of highly defective nickel hydroxide nanostructures is believed to play the decisive role in promoting the dissociative adsorption of water molecules and subsequent oxidative removal of carbonaceous poison on neighbouring platinum sites. As a result, the ternary hybrids exhibit exceptional activity and durability towards efficient methanol oxidation reaction. Under periodic reactivations, the hybrids can endure at least 500,000 s with negligible activity loss, which is, to the best of our knowledge, two to three orders of magnitude longer than all available electrocatalysts.

## Abstract (web summary)

Efficient electrocatalysts are critical for the development of methanol fuel cell technology but most catalysts reported to date suffer from low durability. Here, the authors report platinum-nickel hydroxide-graphene ternary hybrid catalysts with durability extended by two to three orders of magnitude.

## Graphical abstract

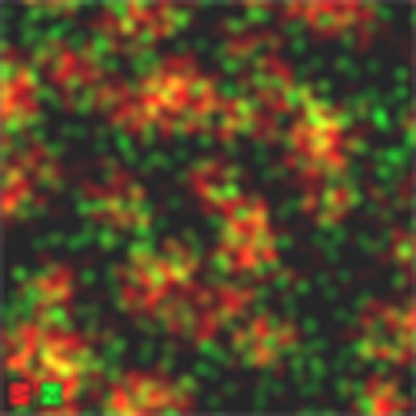

Direct methanol fuel cells (DMFCs) have long been considered as promising power conversion devices for portable and mobile applications^1^,^2^. However, their commercial success is yet to be proven and will largely hinge on two factors: significant reduction of the high cost associated with precious metal electrocatalysts (for example, Pt) and concurrent substantial enhancement of the anode operation durability^1^,^2^,^3^. Over the past several decades, although there has been encouraging progress on the former factor through nanostructuring Pt to increase its mass activity, allowing the use of lower catalyst loadings, the efforts invested on the latter have been far less successful^1^,^2^,^3^,^4^. In the anode, Pt electrocatalysts for methanol oxidation reaction (MOR) are highly susceptible to poisoning by surface-adsorbed reaction intermediates such as CO, leading to quick loss of electrocatalytic activity, sometimes within hundreds of seconds^1^,^2^,^3^,^4^,^5^,^6^. A conventional wisdom to tackle this problem is via alloying Pt with oxophilic metals, Ru being the most successful, to form binary alloys^2^,^3^,^6^. During MOR electrocatalysis, Ru assists in the dissociative adsorption of water molecules to form OH adspecies on its surface. These OH adspecies then promote the oxidation of poisoning CO on neighbouring Pt sites and thereby facilitate their regeneration for further methanol oxidation^2^,^3^,^6^,^7^. Through such a bifunctional mechanism, PtRu binary alloys have been shown to exhibit improved electrocatalytic activity, but they offer very limited gain in durability^8^,^9^. Recent attention has shifted towards the synergy between Pt and a large spectrum of non-precious metal oxides across the periodic table^10^,^11^,^12^,^13^,^14^,^15^,^16^,^17^,^18^. Unfortunately, only incremental improvements in durability have been achieved so far, presumably due to the limited capability of the metal oxides to assist in the oxidative removal of CO on adjacent Pt sites.

Compared with metal oxides, Ni(OH)_2_ is known to better facilitate water dissociation in alkaline electrolytes^19^,^20^. Ni(OH)_2_ prepared at low temperatures usually has proliferative defects, which are particularly active for the dissociative adsorption of water molecules to form OH adspecies^20^. It is suggested that the interaction between Ni(OH)_2_ and OH adspecies is neither too strong nor too weak, which, according to Sabatier principle, has the highest activity to participate in electrocatalytic reactions^19^. Recently, this exact property has motivated the use of Ni(OH)_2_ to enhance the electrocatalytic activity of Pt and other transition metals for hydrogen evolution reaction^20^,^21^,^22^. The synergy between Pt and Ni(OH)_2_ is also shown to promote CO oxidation via the Langmuir–Hinshelwood pathway, leading to a significant reduction of reaction onset overpotentials^19^. In light of the effects, we propose that the bifunctional interaction between Pt and Ni(OH)_2_ may present a possible solution to the long-standing durability issue in MOR electrocatalysis.

Here we report the design and electrocatalytic performance of Pt-Ni(OH)_2_–graphene ternary hybrids for MOR in alkaline environments. Each component of the hybrids fulfills an important specific role: the metal to serve as the active sites for methanol oxidation; the hydroxide to facilitate the oxidative removal of carbonaceous poisons on adjacent metal sites; and the graphene to provide high electric conductivity needed for fast electrocatalysis. These three components have strong mutual interactions and collectively they enable remarkable activity and unprecedented durability for MOR.

## Results

### Synthesis of hybrid electrocatalysts

**Figure 1:**
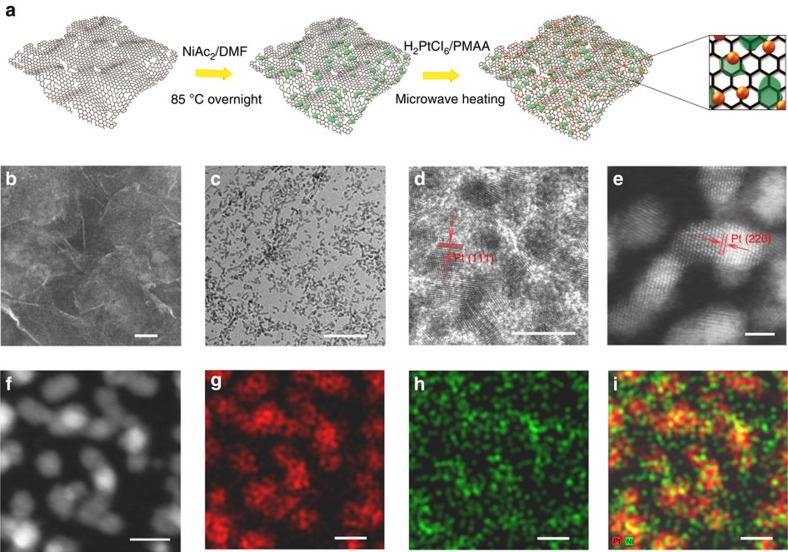
**Preparation and microscopic characterizations of Pt/Ni(OH)**_**2**_**/rGO ternary hybrids.** (**a**) A schematic illustration of the two-step solution method to prepare the ternary hybrid materials. Representative (**b**) SEM, (**c**,**d**) TEM and (**e**) annual dark-field image under STEM (STEM-ADF) images of Pt/Ni(OH)_2_/rGO-4. Representative (**f**) STEM image and its corresponding (**g**) Pt EDS mapping, (**h**) Ni EDS mapping and (**i**) combined Pt and Ni mapping of Pt/Ni(OH)_2_/rGO-4. Microscopic characterization results suggest that small-sized Pt nanocrystals are adequately interfaced with defective Ni(OH)_2_ and supported on rGO nanosheets. Scale bars, 100 nm (**b**), 20 nm (**c**), 2 nm (**d**–**e**) and 4 nm (**f**–**i**).

Pt-Ni(OH)_2_–graphene ternary hybrids were prepared via a two-step solution method as schematically illustrated in Fig. 1a. In the first step, Ni(OH)_2_ nanoparticles were grown on graphene oxide (GO) nanosheets suspended in *N*, *N*-dimethyl formamide solution through controlled hydrolysis of nickel acetate (NiAc_2_) at 85 °C overnight^23^,^24^. Thus, obtained materials were collected, re-dispersed in ethylene glycol, mixed with hexachloroplatinic acid (H_2_PtCl_6_) and the sodium salt of poly(methacrylic acid), and then subjected to intensive microwave heating (see Supplementary Methods for details)^25^. Within 90 s of reaction, H_2_PtCl_6_ was fully reduced to metallic Pt nanocrystals on nanosheets; concurrently, GO was also reduced to form reduced GO (rGO). Following this general protocol, a series of ternary hybrids having varying relative amounts of the three different components were prepared by tuning the starting molar ratios of Pt, Ni and GO precursors. They are denoted as Pt/Ni(OH)_2_/rGO-1–5 with increasing Pt content and decreasing Ni content (Supplementary Table 2). As demonstrated later, the best electrocatalyst for MOR is Pt/Ni(OH)_2_/rGO-4, which is found to contain ∼42.5 wt% Pt and 10.5 wt% Ni from inductive coupled plasma—atomic emission spectroscopy.

### Microstructures of hybrid electrocatalysts

We first carried out electron microscopy studies, to interrogate the structure of the hybrid materials. Figure 1b shows the representative scanning electron microscopy (SEM) image of Pt/Ni(OH)_2_/rGO-4. The rGO nanosheets are intimately coated with patches of Ni(OH)_2_ nanoparticles and small Pt nanocrystals. Under transmission electron microscopy (TEM) examination, Pt metal nanocrystals appear as dark particles about 2 nm in size (Fig. 1c,d). They display clear lattice fringes with an interplanar distance of 0.23 nm, in good agreement with Pt (111) lattice planes (Fig. 1d). The atomic structure of Pt nanocrystals is more clearly revealed from its annual dark-field image under scanning transmission electron microscopy (STEM; Fig. 1e). Unfortunately, we were unable to clearly discern Ni(OH)_2_ nanostructures under current TEM or STEM imaging conditions probably owing to their poor crystallinity and low contrast relative to the rGO background. To probe the spatial distribution of both Ni and Pt species on the rGO support, elemental mapping by energy-dispersive spectroscopy (EDS) was performed under STEM (Fig. 1f–i and additional data set in Supplementary Fig. 1). The distribution of the Pt signal (Fig. 1g) corresponds well with the shape of Pt nanocrystals (Fig. 1f). Ni species were also detected and mapped (Fig. 1h). An overlay of both signals unambiguously illustrates that every Pt nanoscrystal is abundantly surrounded by Ni(OH)_2_ (Fig. 1i). In the hybrids with a high Ni content, for example, Pt/Ni(OH)_2_/rGO-1 and -2, it is observed that rGO nanosheets are coated with denser and thicker Ni(OH)_2_ patches, and that a large fraction of Pt nanocrystals directly grow atop the Ni(OH)_2_ (Supplementary Fig. 2). The chemical compositions of the ternary hybrids are also corroborated by X-ray diffraction (XRD) and X-ray photoelectron spectroscopy (XPS) as summarized in the Supplementary Figs 3 and 4.

**Figure 2:**
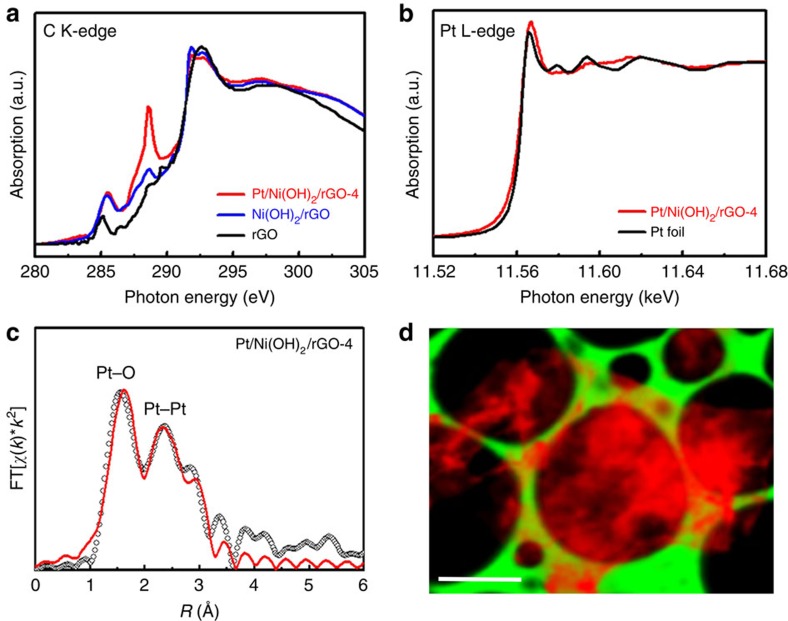
**X-ray absorption spectroscopic characterizations of Pt/Ni(OH)**_**2**_**/rGO ternary hybrids.** (**a**) C K-edge XANES spectrum of Pt/Ni(OH)_2_/rGO-4 in comparison with Ni(OH)_2_/rGO and rGO. (**b**) Pt L_3_-edge XANES spectrum of Pt/Ni(OH)_2_/rGO-4 in comparison with standard Pt foil. (**c**) Fourier transform EXAFS spectrum of Pt/Ni(OH)_2_/rGO-4 and associated fitting curve at the Pt L_3_-edge. (**d**) Representative scanning transmission X-ray microscopy mapping of Ni L_3,2_-edge intensity (red) over one rGO nanosheet suspended on lacy carbon support (green). Scale bar, 2 μm (**d**). XAS data suggest strong mutual interactions between the three components in the hybrid materials.

To investigate the interactions between the different components, we carried out X-ray absorption spectroscopy measurements of the hybrid materials (Fig. 2). Compared with pure rGO nanosheets, the carbon K-edge X-ray absorption near-edge structure (XANES) spectra of Ni(OH)_2_/rGO-4 and Pt/Ni(OH)_2_/rGO-4 show blue-shifted and heightened π* peaks in the range of 285–286 eV (Fig. 2a). These features are robust and reflect the greater structural disorder (less delocalized *π* orbital) of rGO in hybrids and possible electron transfer from rGO to Ni(OH)_2_ or Pt through their interactions, resulting in more C 2*p*_*π*_ unoccupied states^26^. Furthermore, both Ni(OH)_2_/rGO-4 and Pt/Ni(OH)_2_/rGO-4 are characterized with increased peak intensity at 288.6 eV (much more intensity for the ternary hybrid), assignable to carbon atoms in graphene attached to oxygen or other species. This strongly suggests the covalent coupling between rGO and Ni(OH)_2_ or Pt via C–O–Ni or C–O–Pt bonds, respectively^26^,^27^,^28^,^29^. In particular, a large population of C–O–Pt bonds can be inferred from the remarkable rise in peak intensity on the incorporation of Pt nanocrystals into the hybrids.

Interesting results are also garnered from Pt L_3_-edge XANES spectra (Fig. 2b). It is worth noting that the white line intensity of Pt/Ni(OH)_2_/rGO-4 is markedly larger than that of Pt foil. This can be interpreted as the result of the considerable surface oxidation of Pt nanocrystals and, probably, the strong interaction between rGO and Pt nanocrystals^30^,^31^. Figure 2c represents the Fourier-transformed extended X-ray absorption fine structure (FT-EXAFS) together with the theoretical best fit for Pt/Ni(OH)_2_/rGO-4. The FT-EXAFS displays two most intense features, with the one at shorter distance corresponding to Pt–O bonding and the other corresponding to Pt–Pt bonding in the first coordination shell. The pronounced Pt–O contribution again evidences a high degree surface oxidation of Pt and is consistent with the C–O–Pt bonding model deduced from the C K-edge XANES. Fitting of the FT-EXAFS yields an average Pt–Pt coordination number (CN) of 5±1 (Supplementary Fig. 5), which differs substantially from bulk Pt metal (CN=12), owing to the small size of the Pt nanocystals^31^. Similarly, Ni L_3,2_-edge XANES and FT-EXAFS were also collected and are presented in Supplementary Fig. 5. The small CN determined for Ni–Ni (CN∼2 compared with CN=6 for bulk crystalline Ni(OH)_2_) is diagnostic of small, highly defective and poorly crystalline Ni(OH)_2_ nanostructures in the ternary hybrids. In addition, by monitoring the relative intensity of Ni L_3,2_-edge signal under scanning transmission X-ray microscopy, we managed to map out the global distribution of Ni(OH)_2_ and found it to be ubiquitous over the entire rGO nanosheet (Fig. 2d). This microscaled imaging technique nicely complements the STEM–EDS imaging at the nanoscale and underlines the uniform composition of the ternary hybrids.

### MOR activity of hybrid electrocatalysts

**Figure 3:**
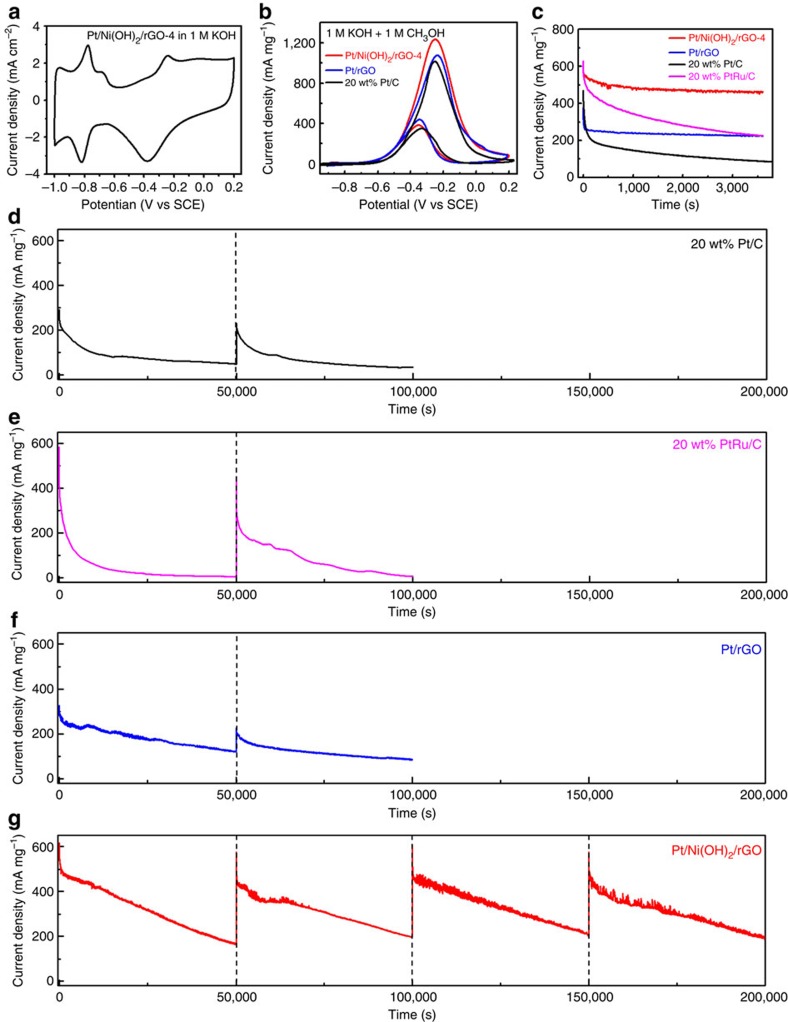
**Electrochemical performance of Pt/Ni(OH)**_**2**_**/rGO ternary hybrids for MOR electrocatalysis.** (**a**) CV curve of Pt/Ni(OH)_2_/rGO-4 in 1 M KOH. (**b**) CV curves of Pt/Ni(OH)_2_/rGO-4, Pt/rGO hybrid and standard 20 wt% Pt/C in 1 M methanol/1 M KOH. (**c**) Short-term durability measurement of Pt/Ni(OH)_2_/rGO-4 at −0.30 V versus SCE in 1 M methanol/1 M KOH in comparison with Pt/rGO, standard 20 wt% Pt/C and 20 wt% PtRu/C. (**d**–**g**) Long-term durability measurements of (**d**) standard 20 wt% Pt/C, (**e**) standard 20 wt% PtRu/C, (**f**) Pt/rGO and (**g**) Pt/Ni(OH)_2_/rGO-4. The dash lines indicate when electrocatalysts were reactivated in clean KOH. In **b**–**g**, current densities are normalized to the mass of precious metals (Pt or Ru) in the working electrode. These electrochemical data suggest high MOR activity and unprecedented durability of Pt/Ni(OH)_2_/rGO hybrids.

Given the above microscopic and spectroscopic results, the next question is whether the interactions among these three components can have a profound synergistic impact on the electrochemical performances of the hybrid materials. This was accomplished by assessing their MOR activities in alkaline electrolytes. Before performing MOR electrocatalysis, electrocatalysts were first activated in N_2_-saturated 1 M KOH by cyclic voltammetry (CV) until a stable curve shape was developed. Figure 3a illustrates the representative CV curve of Pt/Ni(OH)_2_/rGO-4. From its integrated hydrogen desorption charge in the positive-going potential scan (−0.92∼−0.65 V versus saturated calomel electrode (SCE)), we determine that the electrochemical surface area (ECSA) of Pt nanocrystals in Pt/Ni(OH)_2_/rGO-4 is 64.1 m^2^ g^−1^, which is among the largest values reported in literature for Pt-based materials^32^,^33^,^34^. Pt ECSAs of other Pt/Ni(OH)_2_/rGO hybrids and that of the standard 20 wt% Pt/C were likewise calculated and are tabulated in the Supplementary Table 2. As expected, the hybrids with higher Ni(OH)_2_ contents have smaller ECSAs, as in these materials a large fraction of Pt nanocrystals are grown on insulating Ni(OH)_2_ and do not actively engage in electrochemical reactions. Standard 20 wt% Pt/C is measured to have an ECSA of 36.1 m^2^ g^−1^.

In the presence of 1 M methanol, the CV curves are dramatically modified (Fig. 3b and Supplementary Fig. 6). They display an intense anodic peak in the forward scan and a minor anodic peak in the reverse scan over the potential range of −0.6∼0.0 V—this contribution derives from the MOR catalysed by Pt. Insights into MOR activity can be gained by quantitatively comparing their initial peak current densities in the forward scan. Among all the electrocatalysts examined, Pt/Ni(OH)_2_/rGO-4 demonstrates the highest peak current density of 1,236 mA mg^−1^_Pt_, slightly larger than both the 20 wt% Pt/C (1,016 mA mg^−1^_Pt_) and Pt/rGO (1,076 mA mg^−1^_Pt_). For the ternary hybrids, Pt/Ni(OH)_2_/rGO-1–5 all exhibit significant peak current densities in the range of 800–1,000 mA mg^−1^_Pt_, larger than most reported values in literatures (Supplementary Table 3).

### MOR durability of hybrid electrocatalysts and their reactivations

One problem plaguing the development of MOR electrocatalysts is their poor operation durability^3^,^6^. Irrespective of their initial activities, all previous electrocatalysts are subject to quick activity loss and become largely inactive sometimes within several hundreds of seconds (Supplementary Table 3). It is therefore one of the main pursuits of this study to scrutinize the durability of our ternary hybrids. As a starting point, we screened their short-term operation durability. Chronoamperometric (*i*∼*t*) responses of different electrocatalysts at the potential of −0.3 V versus SCE were measured for 1 h (Fig. 3c and Supplementary Fig. 6). The 20 wt% Pt/C benchmark shows a rapid initial decay, losing >50% of its initial current density in <200 s. At the end of the experiment, only <20% of initial current density is retained, in good agreement with previous observations^35^. Alloying Pt with Ru in 20 wt% PtRu/C improves initial activity but fails to alleviate its poor durability. When Pt nanocrystals are directly grown on rGO nanosheets, their MOR activity is barely improved but durability significantly rises with ∼220 mA mg^−1^_Pt_ retained after 1 h. The positive effect of rGO on the short-term durability of Pt was previously attributed to the synergistic coupling between rGO and Pt^35^,^36^,^37^,^38^. Impressively, Pt/Ni(OH)_2_/rGO-4 demonstrates both remarkable activity and stability. Its current density after 1 h of operation retains >460 mA mg^−1^_Pt_. As far as we know, this value represents exceptional short-term stability, exceeding those of all MOR electrocatalysts available (Supplementary Table 3). Among other ternary hybrids, Pt/Ni(OH)_2_/rGO-3 and 5 exhibit slightly inferior performances, while Pt/Ni(OH)_2_/rGO-1 and 2 are not as appealing due to their higher Ni(OH)_2_ content (Supplementary Fig. 6).

We next evaluated the long-term operation durability of different electrocatalysts (Fig. 3d–g). At the end of 50,000 s continuous operation, negligible activities remain for 20 wt% Pt/C and 20 wt% PtRu/C (Fig. 3d,e). In stark contrast, Pt/rGO and Pt/Ni(OH)_2_/rGO-4 still deliver a current density of 120 and 160 mA mg^−1^_Pt_, respectively. Even though this result is already quite successful, we further note that the observed activity loss stems from slow but inevitable poisoning of the electrocatalysts. One strategy to further enhance durability is to force electrolyte circulation that continuously removes the reaction byproducts and slows down the poisoning process. For example, using our own customized flow cell, we record that Pt/Ni(OH)_2_/rGO-4 can reproducibly retain a current density of ∼400 mA mg^−1^_Pt_ after 80,000 s continuous operation (Supplementary Fig. 7). Such durability is unprecedented (about one to approximately two orders magnitude longer than all literature values, see Supplementary Table 3) and is yet still far from the limit that our ternary hybrid electrocatalyst can possibly endure.

Even more exciting, we find that Pt/Ni(OH)_2_/rGO-4 can be simply reactivated by a few CV cycles in 1 M KOH after the 50,000 s durability measurement to recover full MOR activity to the initial value and then is ready for another 50,000 s continuous operation in a fresh methanol electrolyte (Fig. 3g). This observation is highly reproducible. We repeat the cycling up to ten times for a total of 500,000 s and every time full activity is recovered after reactivation (Supplementary Fig. 8). This property is unique to the ternary hybrids. In contrast, although Pt/rGO exhibits excellent stability in the first 50,000 s operation, its current density can only be partially recovered during the same reactivation procedure. Moreover, in the subsequent cycle the recovered activity is quickly lost and the current density falls back to its declining trajectory of the previous cycle (Fig. 3f). Analysis of reactivated electrocatalysts reveals that Pt/Ni(OH)_2_/rGO-4 retains ∼74% of its original ECSA after the first 50,000 s, larger than both 20 wt% Pt/C (∼62%) and Pt/rGO (∼69%; Supplementary Fig. 9 and Supplementary Tables 1 and 2). TEM examinations of the three catalysts suggest that after the long durability test, some Pt nanocrystals aggregate to form elongated shapes, in line with their reduced ECSA (Supplementary Fig. 10). When subjected to CV measurements in a fresh methanol electrolyte, the reactivated Pt/Ni(OH)_2_/rGO-1–5 demonstrate negligible loss of peak current densities, whereas decays are more pronounced for 20 wt% Pt/C, 20 wt% PtRu/C and Pt/rGO (Supplementary Fig. 11). In real DMFC devices, it would be highly desirable if MOR electrocatalysts could be reactivated during operation breaks by simply switching the electrolyte to clean KOH solution and running a few CV cycles. The reactivation could be done at a reasonable frequency—for example, once per day, similar to the frequency of battery recharging—to enable a prolonged electrocatalyst lifetime.

## Discussion

**Figure 4:**
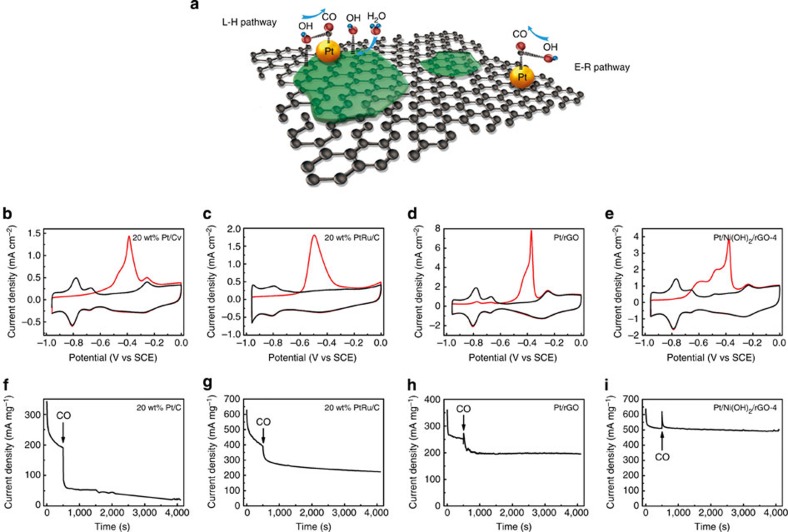
**The critical role of defective Ni(OH)**_**2**_ **to the exceptional MOR performance of ternary hybrids.** (**a**) A schematic illustration showing the bifunctional interaction between Ni(OH)_2_ and adjacent Pt sites for the dissociate adsorption of water molecules and subsequently the oxidative removal of CO on Pt sites via the L–H pathway. CO stripping experiments of (**b**) standard 20 wt% Pt/C, (**c**) standard 20 wt% PtRu/C, (**d**) Pt/rGO and (**e**) Pt/Ni(OH)_2_/rGO-4 in 1 M KOH. (**f**–**i**) Response of (**f**) standard 20 wt% Pt/C, (**g**) standard 20 wt% PtRu/C, (**h**) Pt/rGO and (**i**) Pt/Ni(OH)_2_/rGO-4 to intentional CO poisoning in 1 M methanol/1 M KOH. During the chronoamperometric measurements at −0.3 V, 10% CO/N_2_ gas was bubbled to the electrolyte at where arrows indicate. The incorporation of defective Ni(OH)_2_ enables high CO resistance and superb MOR performance of ternary hybrids.

Through the above investigations, we have established that the ternary hybrid electrocatalysts, especially Pt/Ni(OH)_2_/rGO-4, have superb activity and stability for the MOR in alkaline electrolytes. The presence of defective Ni(OH)_2_ is the critical key to their outstanding performances. In an alkaline environment, the rate-determining step of MOR on Pt is the oxidative removal of surface adsorbed carbonaceous intermediates, for example, CO, with the assistance of OH^2^,^7^. Free OH^−^ ions in alkaline electrolytes could participate in this reaction via the Eley–Rideal mechanism (Fig. 4a)^39^; however, a more predominant pathway is suggested to be reactions with OH adspecies either on or around Pt sites via the Langmuir–Hinshelwood mechanism^39^,^40^. If sufficient OH adspecies are supplied nearby, the rate-determining step can be greatly accelerated, and so this forms the basis for designing Pt alloys (including PtRu) and Pt–metal oxide hybrids for MOR electrocatalysis^3^,^6^. Compared with metals or metal oxides, Ni(OH)_2_ is particularly active for the dissociative adsorption of water molecules to form OH adspecies due to its suitable bonding strength with OH^19^,^20^. Even though there are a handful of reported activities of PtNi alloy, Pt–Ni or Pt–NiO hybrid electrocatalysts (mostly with incremental performances)^11^,^39^,^41^,^42^, we are not aware of any previous effort to take advantage of the unique bifunctional coupling between Pt and the hydroxide form of Ni for MOR. In this study, highly defective and poorly crystalline Ni(OH)_2_ nanostructures are prepared in intimate contact with small Pt nanocrystals and supported on rGO nanosheets as shown by our detailed structural characterizations. According to prior reports, these defective Ni(OH)_2_ species greatly expedite water dissociation^20^. Subsequent OH spillover from Ni(OH)_2_ to neighbouring Pt nanocrystals provides an efficient channel to supply OH adspecies and promotes the oxidative removal of carbonaceous poisons on Pt in proximity. They are critical to the observed high activity and durability of hybrid electrocatalysts (Fig. 4a)^20^. In addition, our control experiment indicates that the attempt to increase the crystallinity of Ni(OH)_2_ by introducing an additional hydrothermal step during the synthesis of hybrid materials adversely results in inferior electrocatalytic performance (Supplementary Fig. 12).

The benefit of Ni(OH)_2_ on the oxidative removal of CO can be clearly borne out by CO-stripping voltammetry. For the standard 20 wt% Pt/C benchmark catalyst, two oxidation peaks are observed in the first anodic scan over the potential range of −0.5 to approximately−0.3 V versus SCE, due to CO electrooxidation at different lattice sites of Pt nanocrystals (Fig. 4b)^39^. Alloying Pt with Ru in 20 wt% PtRu/C, as expected, promotes CO electroxidation, resulting in reduction of the onset potential to around −0.6 V (Fig. 4c). Most interestingly, in the CO-stripping CVs of Pt/Ni(OH)_2_/rGO, other than the doublet peak shared with both 20 wt% Pt/C and Pt/rGO, a new broad peak emerges at a much negative potential of −0.7 to approximately −0.5 V (Fig. 4e and Supplementary Fig. 13). We attribute the former to CO electroxidation at Pt sites far away from Ni(OH)_2_ in the ternary hybrids and the latter to CO electroxidation at Pt sites in the periphery of Ni(OH)_2_, which is much more facile through bifunctional interaction between Pt and Ni(OH)_2_ as elaborated above. Integration of peak areas suggests that ∼30% of Pt sites fall within the periphery of Ni(OH)_2_ (Supplementary Fig. 14). On these hotspots, CO can be efficiently oxidized and removed at potentials pertinent to MOR electrocatalysis (>−0.5 V). As a result, they are incredibly CO resistant. To further demonstrate this, CO gas was intentionally bubbled to the electrolyte at the middle of a chronoamperometric experiment on Pt/Ni(OH)_2_/rGO-4. As shown in Fig. 4i, no loss in MOR current density can be discerned, in stark contrast to the sharp loss in current density during the same experiment on 20 wt% Pt/C, 20 wt% PtRu/C and Pt/rGO (Fig. 4f–h). Moreover, the hybrid electrocatalyst is found to be resistant to all other possible MOR intermediates and products, that is, formaldehyde, formic acid, methyl formate and K_2_CO_3_ (Supplementary Fig. 15).

Finally, we emphasize the Pt-Ni(OH)_2_–graphene synergy. The three components of the ternary hybrids have drastically different chemical natures and each of them fulfils an important specific role: Pt nanocrystals to serve as the active sites for MOR; Ni(OH)_2_ to facilitate the oxidative removal of carbonaceous poisons from adjacent Pt sites and graphene to provide high electric conductivity needed for fast electrocatalysis and to suppress the aggregation of supported Pt and Ni(OH)_2_. The superb MOR performance is never possible when anyone of them is missing. Furthermore, as corroborated by X-ray absorption spectroscopy experiments, these individual components have strong mutual interactions, which otherwise do not exist in their physical mixtures. Control experiments show that the physical mixture of Pt/Ni(OH)_2_ and rGO or Pt/rGO and Ni(OH)_2_ has much worse MOR activities (Supplementary Fig. 16). We are currently setting up *in-situ* Fourier transform infrared spectroscopy experiments, to monitor the change of Pt surfaces in the ternary hybrids during MOR so as to further understand their superiority over conventional Pt electrocatalysts^43^,^44^.

In summary, we report a two-step solution method to successfully prepare the Pt/Ni(OH)_2_/rGO ternary hybrids. The hybrids are featured with small-sized Pt nanocrystals intimately interfaced with highly defective Ni(OH)_2_ nanostructures and supported on conductive rGO nanosheets. The incorporation of Ni(OH)_2_ greatly facilitates the dissociative adsorption of water molecules and subsequently assists in the oxidative removal of carbonaceous poison via the Langmuir–Hinshelwood reaction pathway. Through collective synergy, the three functional components of the hybrid materials together achieve impressive MOR activity and durability far better than those previously reported. Long-term operation durability measurements demonstrate that the best ternary hybrid, under continuous electrolyte circulation, can sustain a current density of ∼400 mA mg^−1^_Pt_ even after 80,000 s operation and, via periodic reactivations, can endure at least 500,000 s with negligible activity loss. The unprecedented performances of the hybrids for MOR electrocatalysis represent an important step forward towards their commercial applications in DMFCs.

## Methods

### Materials synthesis

Pt/Ni(OH)_2_/rGO ternary hybrids were prepared by a two-step solution method. In the first step, a calculated amount of Ni(Ac)_2_ aqueous solution was added into a *N*, *N*-dimethyl formamide dispersion of GO and reacted at 85 °C for 12 h under vigorous magnetic stirring. The resulting Ni(OH)_2_/GO was collected, washed and transferred to an ethylene glycol solution of calculated amounts of H_2_PtCl_6_ and poly(methacrylic acid). The suspension was then subjected to microwave heating at 800 W for 90 s. Final products were collected by centrifugation, repetitively washed and lyophilized. Please refer to Supplementary Methods for detailed experimental procedures.

### Characterization

SEM images were taken from Zeiss scanning electron microscope. TEM was conducted on FEI Tecnai G^2^ F20 TEM at an acceleration voltage of 200 kV. STEM–EDS characterization was carried out using a FEI Titan G^2^ ChemiSTEM operated at an acceleration voltage of 200 kV. XRD was performed on PANalytical X-ray diffractometer. XPS spectra were collected on SSI S-Probe XPS Spectrometer. Inductive coupled plasma—atomic emission spectroscopy measurements were conducted on Varian Vista MPX; samples were first calcined in air at 600 °C for 30 min, then digested in concentrated HNO_3_ and diluted to desired concentrations.

### Electrochemical measurements

For CV and chronoamperometric measurements, 4 mg of catalysts and 24 μl of 5 wt% Nafion solution were added to 0.50 ml of ethanol and 0.50 ml of H_2_O, and vigorously sonicated for >30 min to form a homogeneous ink. Then, 5 μl of the catalyst ink (containing 20 μg of catalyst) was loaded onto a glassy carbon electrode of 3 mm in diameter to achieve a loading density of 0.28 mg cm^−2^. Experiments were carried out in a standard three-electrode configuration with the glassy carbon electrode, a saturated calomel electrode and a graphite rod as the working, reference and counter electrode, respectively. The working electrode was first activated in N_2_-saturated 1 M KOH by fast CV cycling for about 50 cycles until the curve stabilizes and then was switched to 1 M methanol/1 M KOH solution for subsequent MOR assessments. For the periodic reactivation of electrocatalysts during long-term durability measurements, the working electrode was switched back to 1 M KOH and reactivated by running fast CV cycling. For CO stripping measurements, a monolayer of CO was adsorbed on electrocatalysts by flowing a 10% CO/N_2_ in 1 M KOH for 30 min, while the electrode was held at −0.96 V. Non-adsorbed CO was removed by bubbling the electrolyte with N_2_ for 15 min before running the stripping experiment. Please refer to Supplementary Methods for detailed experimental procedures.

## Additional information

**How to cite this article:** Huang, W. *et al.* Highly active and durable methanol oxidation electrocatalyst based on the synergy of platinum–nickel hydroxide–graphene. *Nat. Commun.* 6:10035 doi: 10.1038/ncomms10035 (2015).

## Supplementary Material

Supplementary Information Supplementary Figures 1-16, Supplementary Tables 1-3, Supplementary Methods and Supplementary References
