# Supplementary material for: Highly active and durable methanol oxidation electrocatalyst based on the synergy of platinum–nickel hydroxide–graphene
Source: Nat Commun. 2015 Nov 25;6:10035. doi: 10.1038/ncomms10035 (PMC4674678; doi:10.1038/ncomms10035)
Supplement: Supplementary Information — Supplementary Figures 1-16, Supplementary Tables 1-3, Supplementary Methods and Supplementary References [file ncomms10035-s1.pdf]

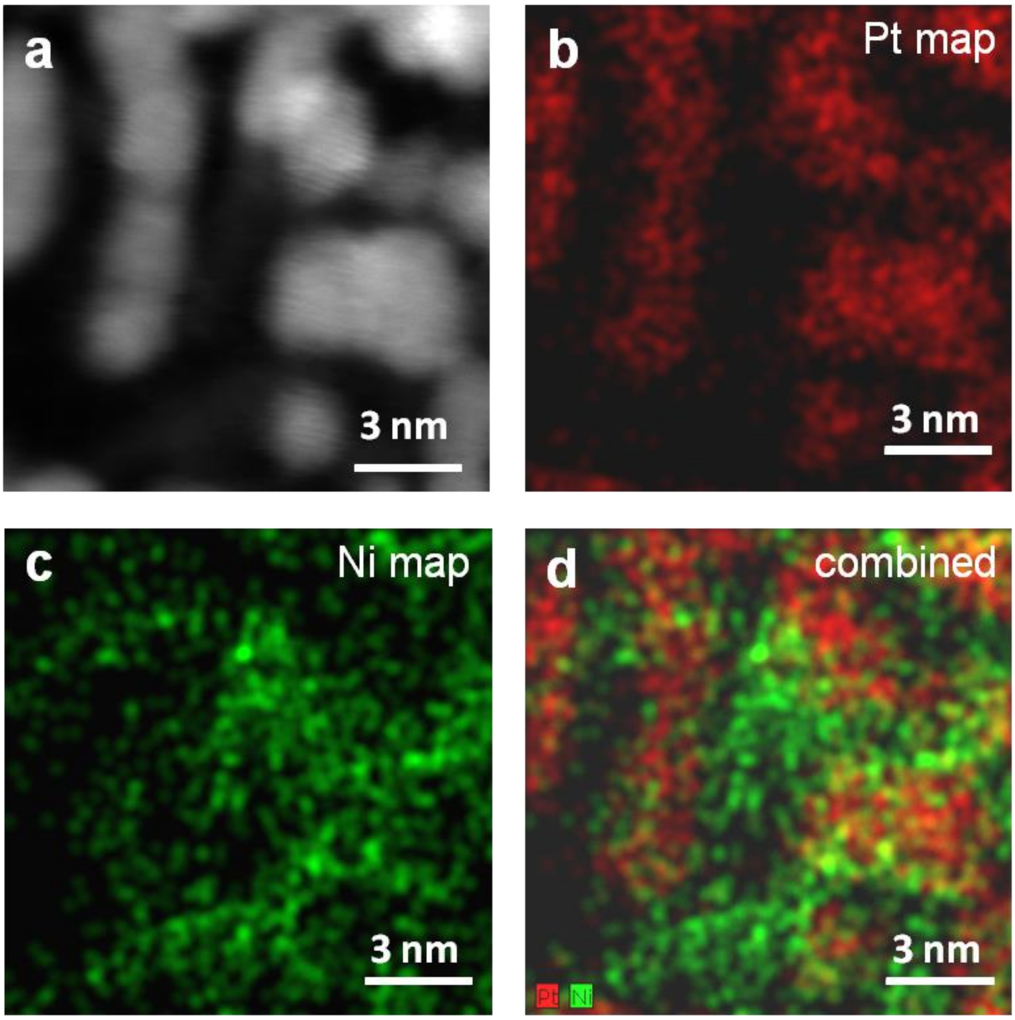

Supplementary Figure 1. (a) STEM image of Pt/Ni(OH)$_2$/rGO-4 and its corresponding (b) Pt EDS mapping, (c) Ni EDS mapping and (d) combined Pt and Ni mapping.

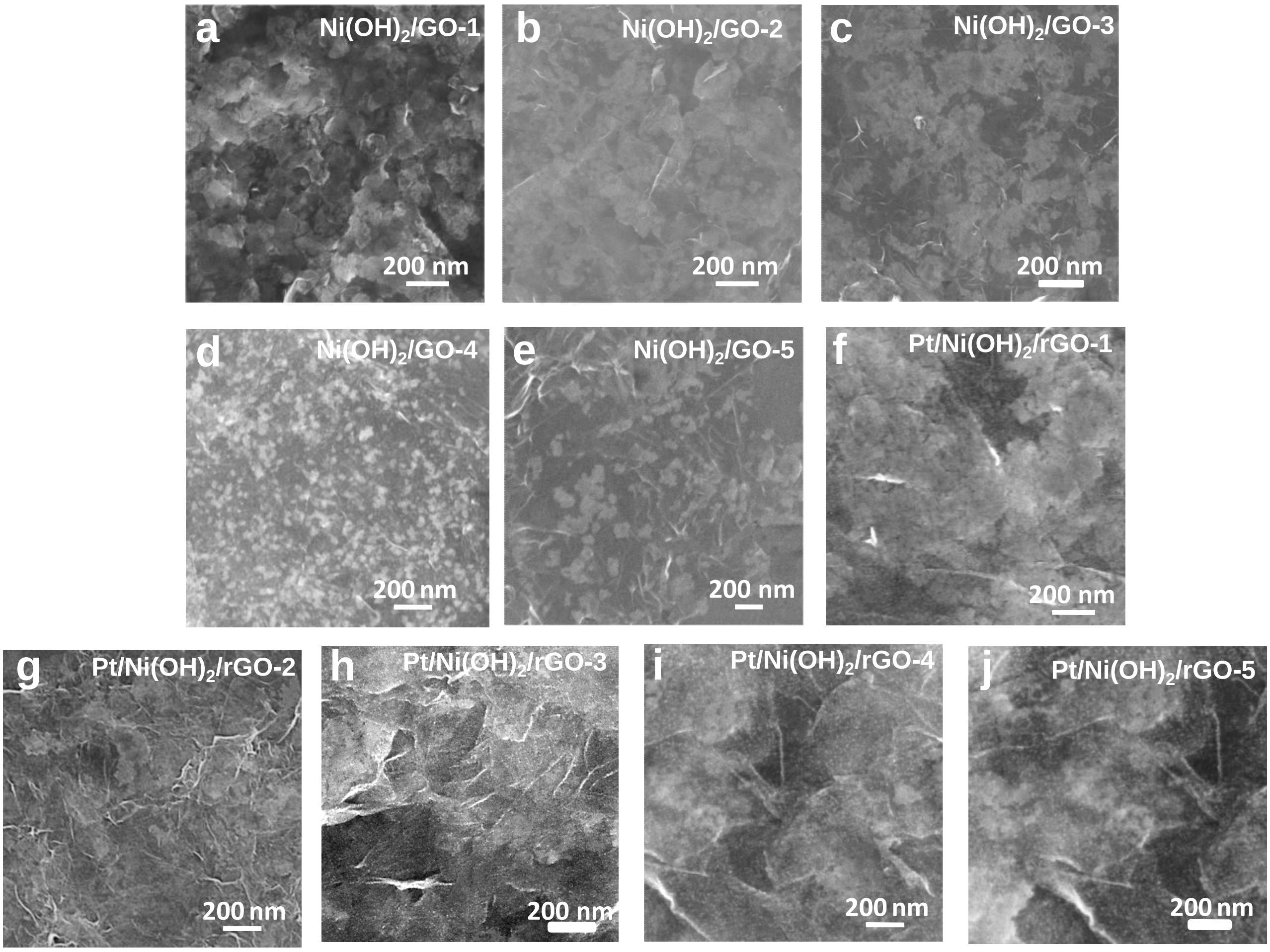

Supplementary Figure 2. SEM images of (a-e) $Ni(OH)_2$/GO-1 to 5 from the first step and (f-j) Pt/$Ni(OH)_2$/rGO-1 to 5 final products.

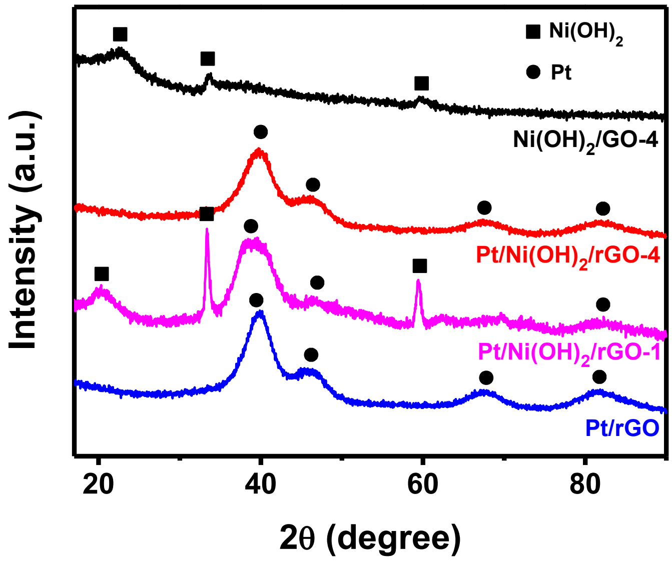

Supplementary Figure 3. XRD patterns of $Ni(OH)_2$/GO-4, Pt/$Ni(OH)_2$/rGO-4, Pt/$Ni(OH)_2$/rGO-1 and Pt/rGO. Diffraction peaks from $Ni(OH)_2$ and metallic Pt are marked with squares and circles, respectively.

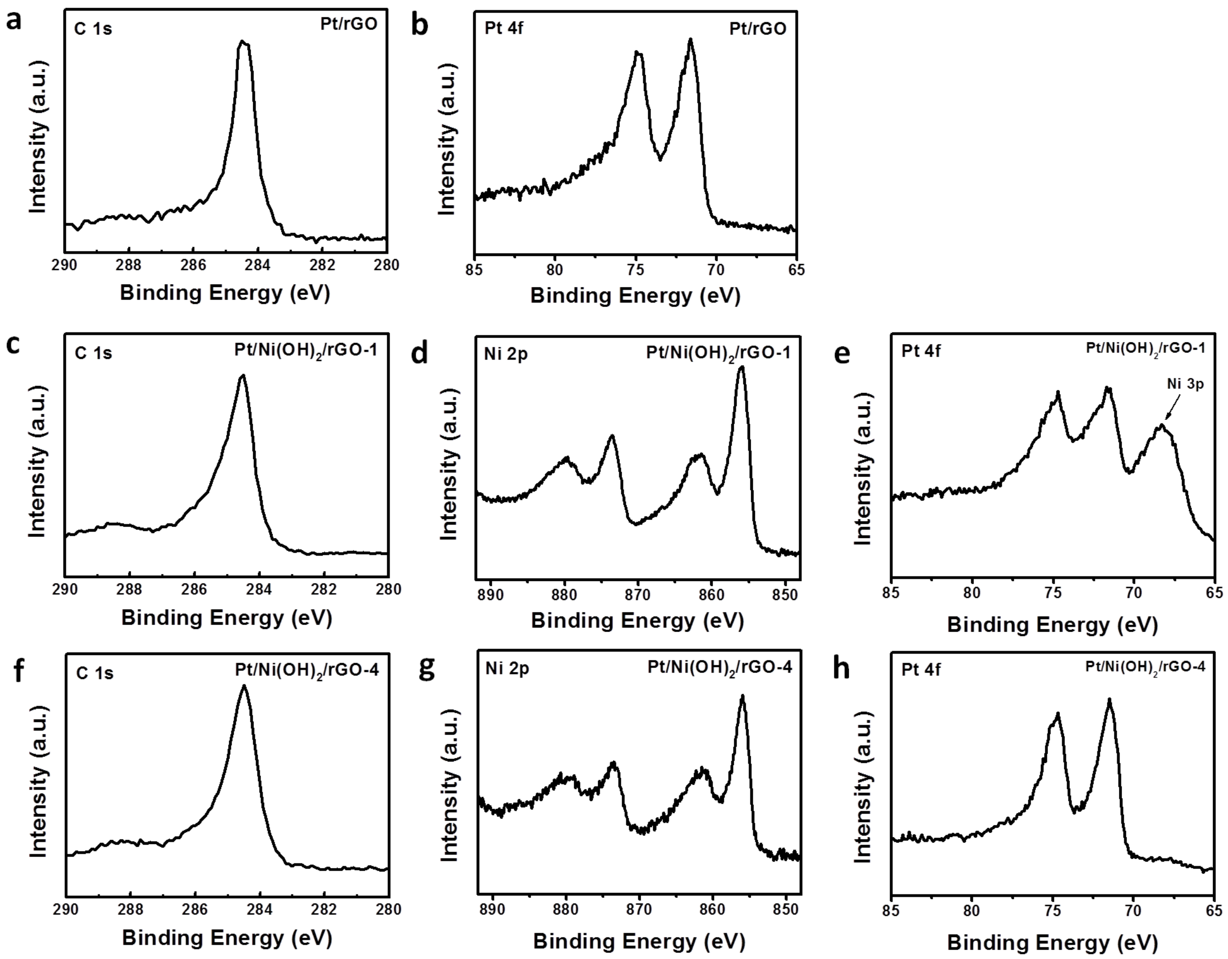

Supplementary Figure 4. High-resolution C 1s, Ni 2p and Pt 4f XPS spectra of (a-b) Pt/rGO, (c-e) Pt/$Ni(OH)_2$/rGO-1, and (f-h) Pt/$Ni(OH)_2$/rGO-4.

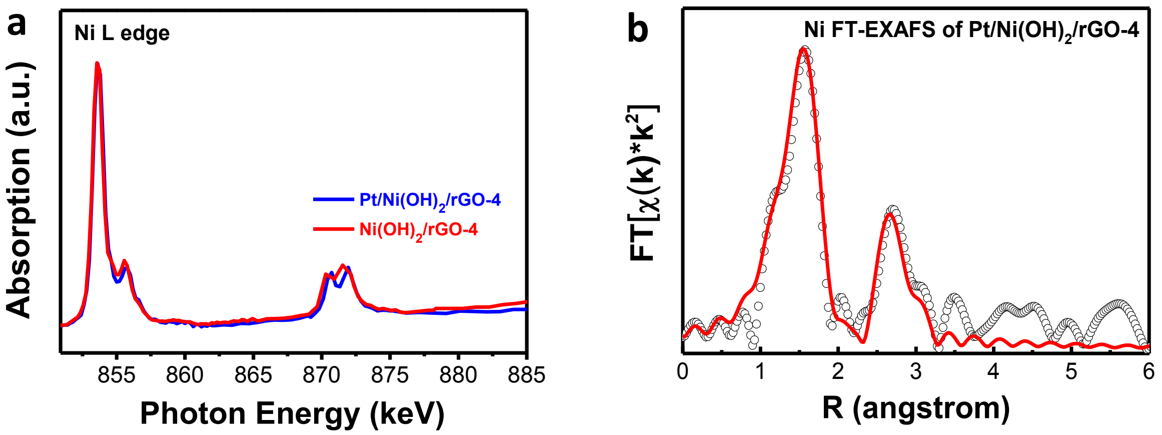

c	Path	Coordination Number	Bond Length / Å	σ^2 / 10^{-3} Å^2	ΔE_0 / eV
$Ni(OH)_2$/rGO-4	Ni–O	6	2.055(7)	7.3(5)	-4.4(8)
	Ni–Ni	4.8(8)	3.099(6)	7(1)	
Pt/$Ni(OH)_2$/rGO-4	Ni–O	6	2.04(1)	6.4(9)	-6(2)
	Ni–Ni	2(1)	3.10(2)	3(4)	
Pt/rGO	Pt–O	1.5(4)	1.94(1)	1(3)	-2(2)
	Pt–Pt	4(1)	2.70(2)	8(2)	
Pt/$Ni(OH)_2$/rGO-4	Pt–O	1.4(5)	1.98(2)	3(4)	4(2)
	Pt–Pt	5(1)	2.74(2)	7(2)	

Supplementary Figure 5. (a) Ni $L_{2,3}$-edge XANES spectra of Pt/$Ni(OH)_2$/rGO-4 and $Ni(OH)_2$/rGO-4. (b) Fourier transform EXAFS spectrum of Pt/$Ni(OH)_2$/rGO-4 and associated fitting curve at the Ni K edge. (c) Table summarizing structural parameters obtained from the analysis of the EXAFS spectra of Pt/$Ni(OH)_2$/rGO-4, $Ni(OH)_2$/rGO-4 and Pt/rGO at Ni K edge and Pt L_3 edge.

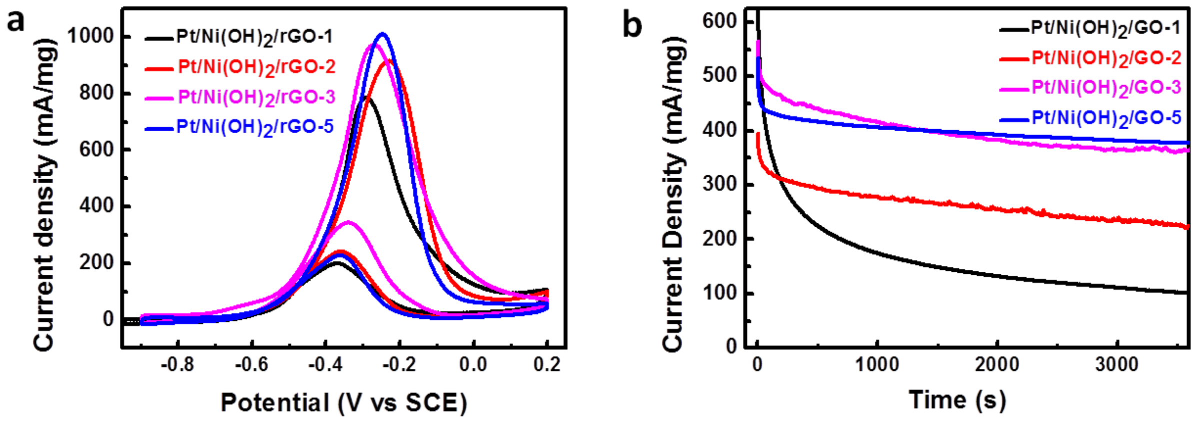

Supplementary Figure 6. (a) MOR CV curves of Pt/Ni(OH)$_2$/rGO-1, -2, -3 and -5 and (b) their corresponding short-term durability measurement at -0.30 V vs. SCE in 1 M methanol/1 M KOH.

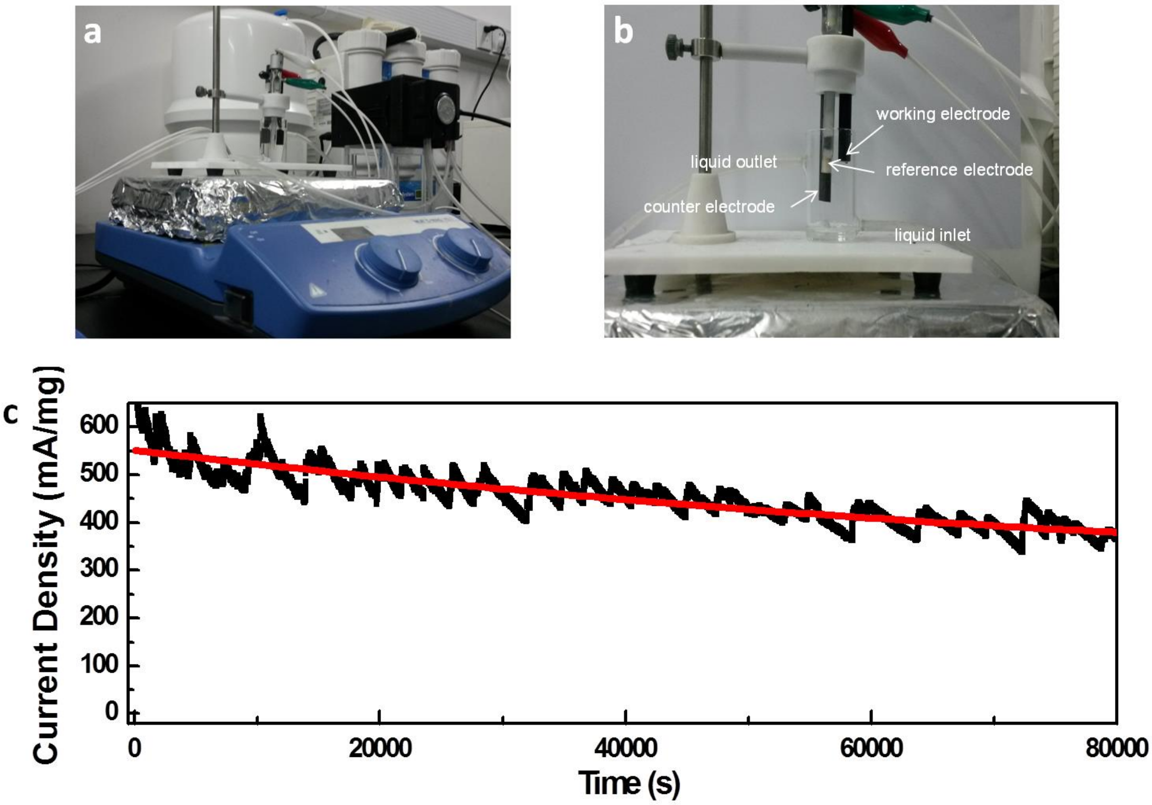

Supplementary Figure 7. (a-b) The setup of our customized flow cell for long-term durability assessments. (b) Chronoamperometry of Pt/Ni$(OH)_2$/rGO-4 for MOR at -0.3 V in the flow cell with continuous electrolyte circulation.

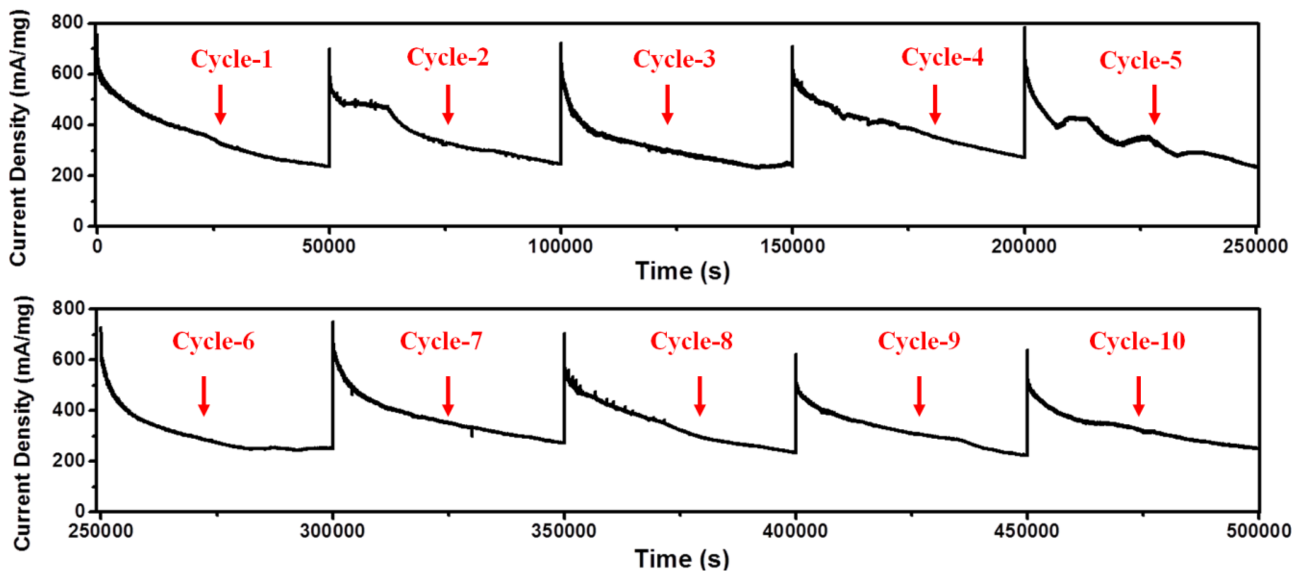

Supplementary Figure 8. Long-term durability measurements of Pt/Ni(OH)$_2$/rGO-4 with periodic reactivations for a total of 10 cycles and a period of 500,000 s.

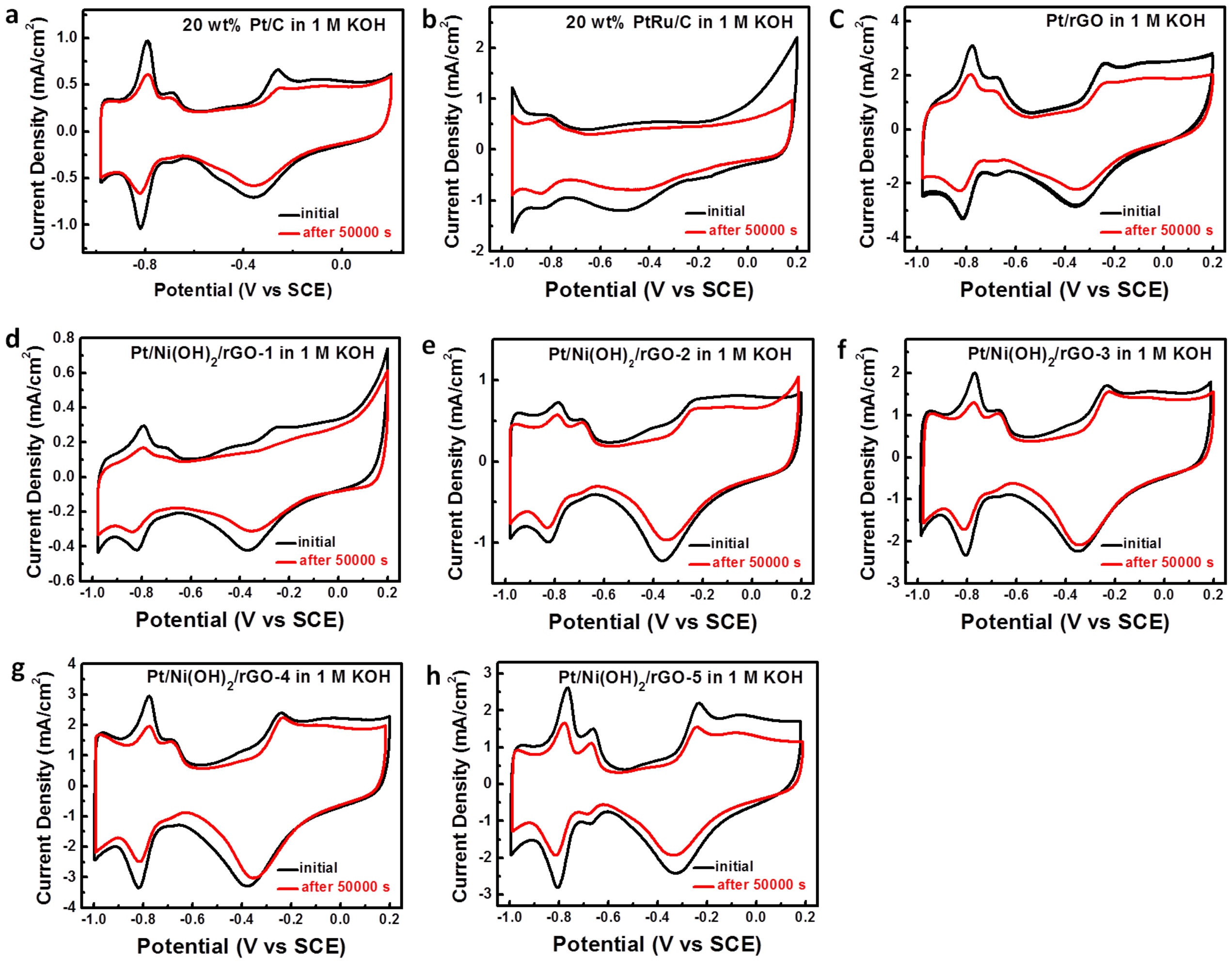

Supplementary Figure 9. CV curves of different electrocatalysts as indicated before (black) and reactivated after (red) 50,000 s MOR electrocatalysis in 1 M KOH. ECSAs of Pt were calculated based on their integrated hydrogen desorption charge in the positive-going potential scan and tabulated in Supplementary Table 1.

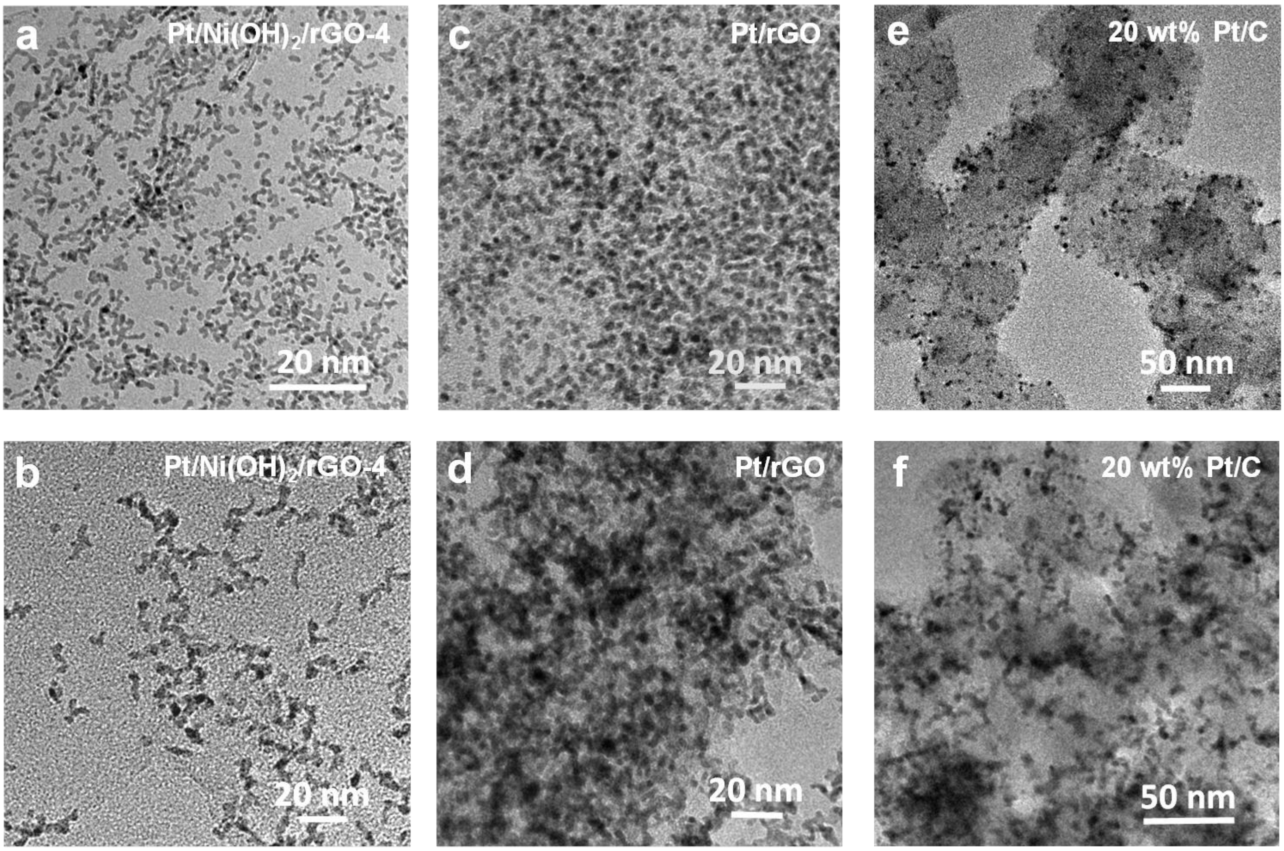

Supplementary Figure 10. TEM images of Pt/Ni(OH)$_2$/rGO-4, Pt/rGO and 20 wt% Pt/C (a,c,e) before and (b,d,f) after the 50,000 s durability test.

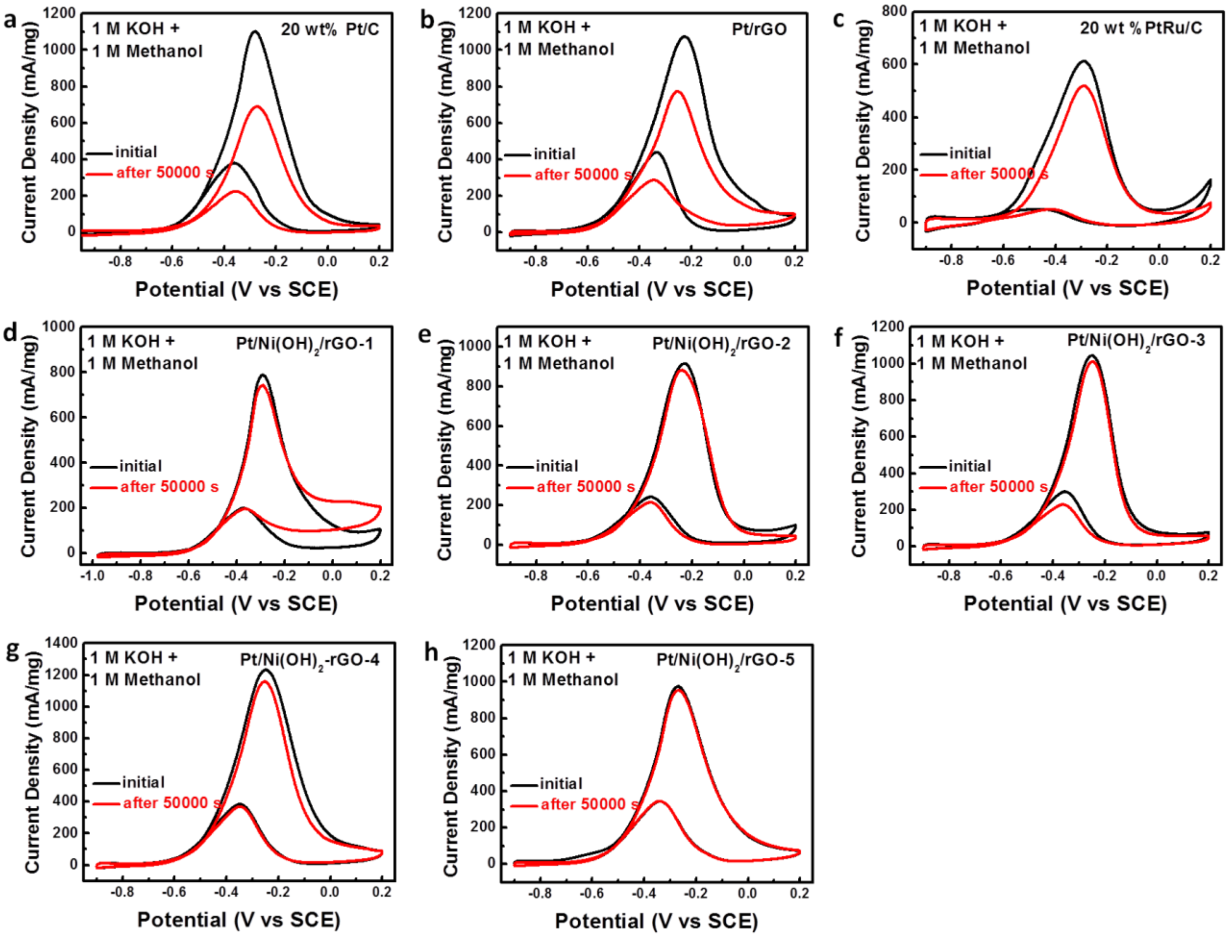

Supplementary Figure 11. MOR CV curves of different electrocatalysts as indicated before (black) and reactivated after (red) 50,000 s MOR electrocatalysis in 1 M methanol/1 M KOH.

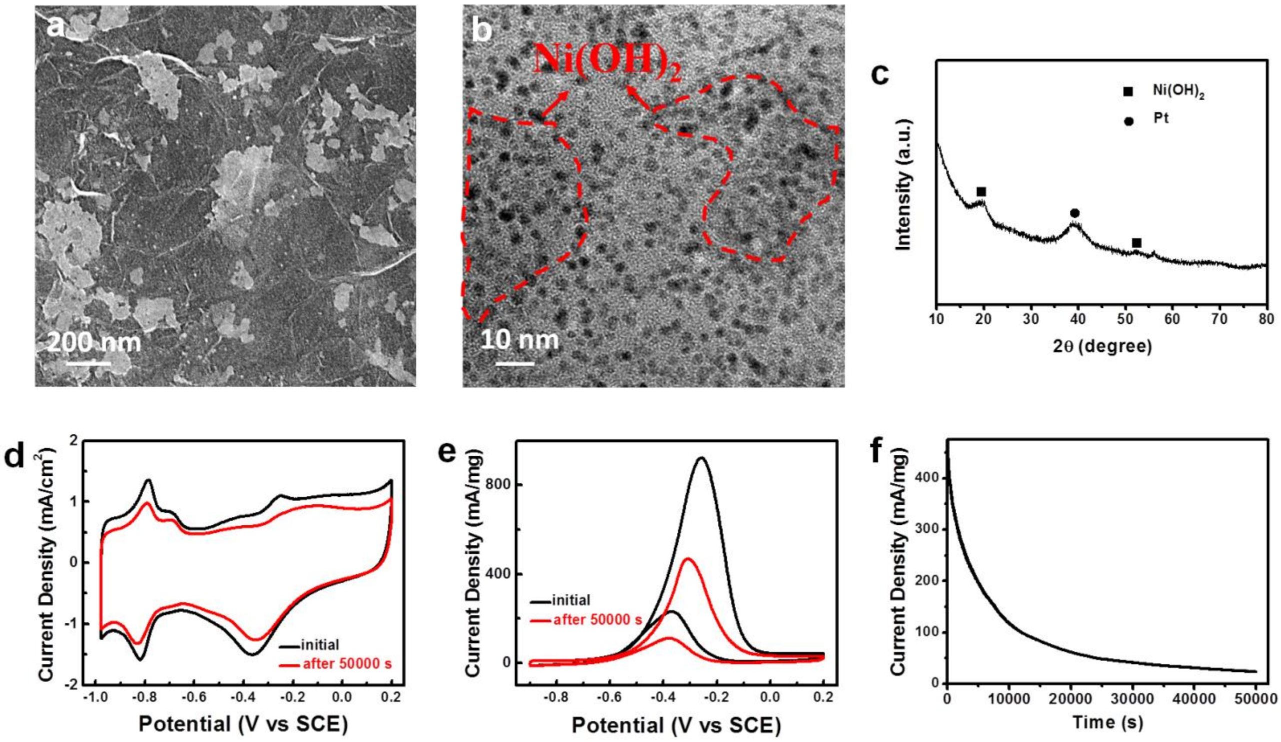

Supplementary Figure 12. (a) SEM image, (b) TEM image and (c) XRD pattern of Pt/ht-$Ni(OH)_2$/rGO-4, and its (d) CV curve in 1 M KOH, (e) CV curve in 1 M methanol/1 M KOH and (f) MOR durability measurement. The data here support that less defective $Ni(OH)_2$ is not as effective as highly defective $Ni(OH)_2$ in promoting MOR electrocatalysis.

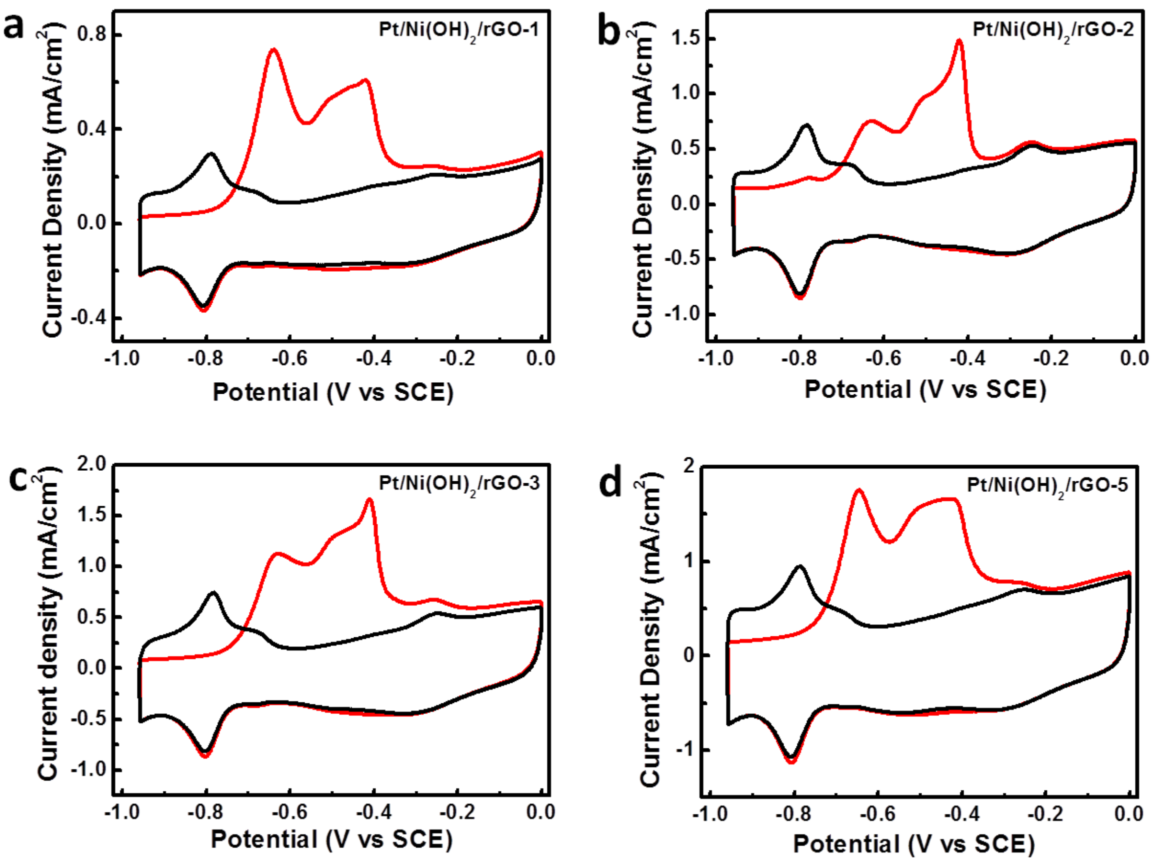

Supplementary Figure 13. CO-stripping voltammetry of (a) Pt/Ni$(OH)_2$/rGO-1, (b) Pt/Ni$(OH)_2$/ rGO-2, (c) Pt/Ni$(OH)_2$/rGO-3 and (d) Pt/Ni$(OH)_2$/rGO-5 in 1 M KOH solution.

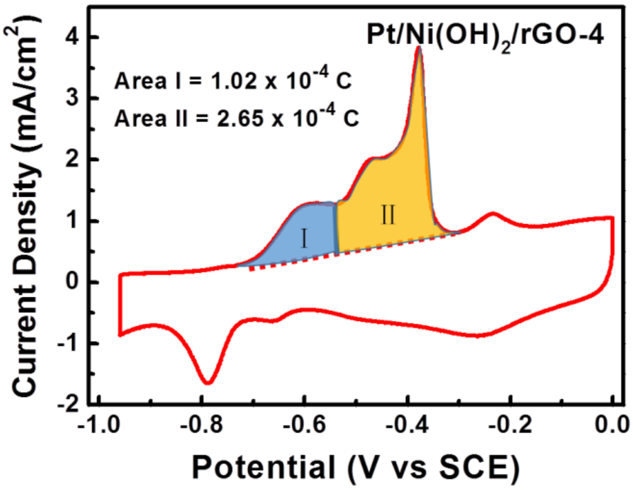

Supplementary Figure 14. CO stripping curve of Pt/$Ni(OH)_2$/rGO-4 with its CO electroxidation peak deconvoluted to reflect contributions from Pt sites close to or far away from $Ni(OH)_2$.

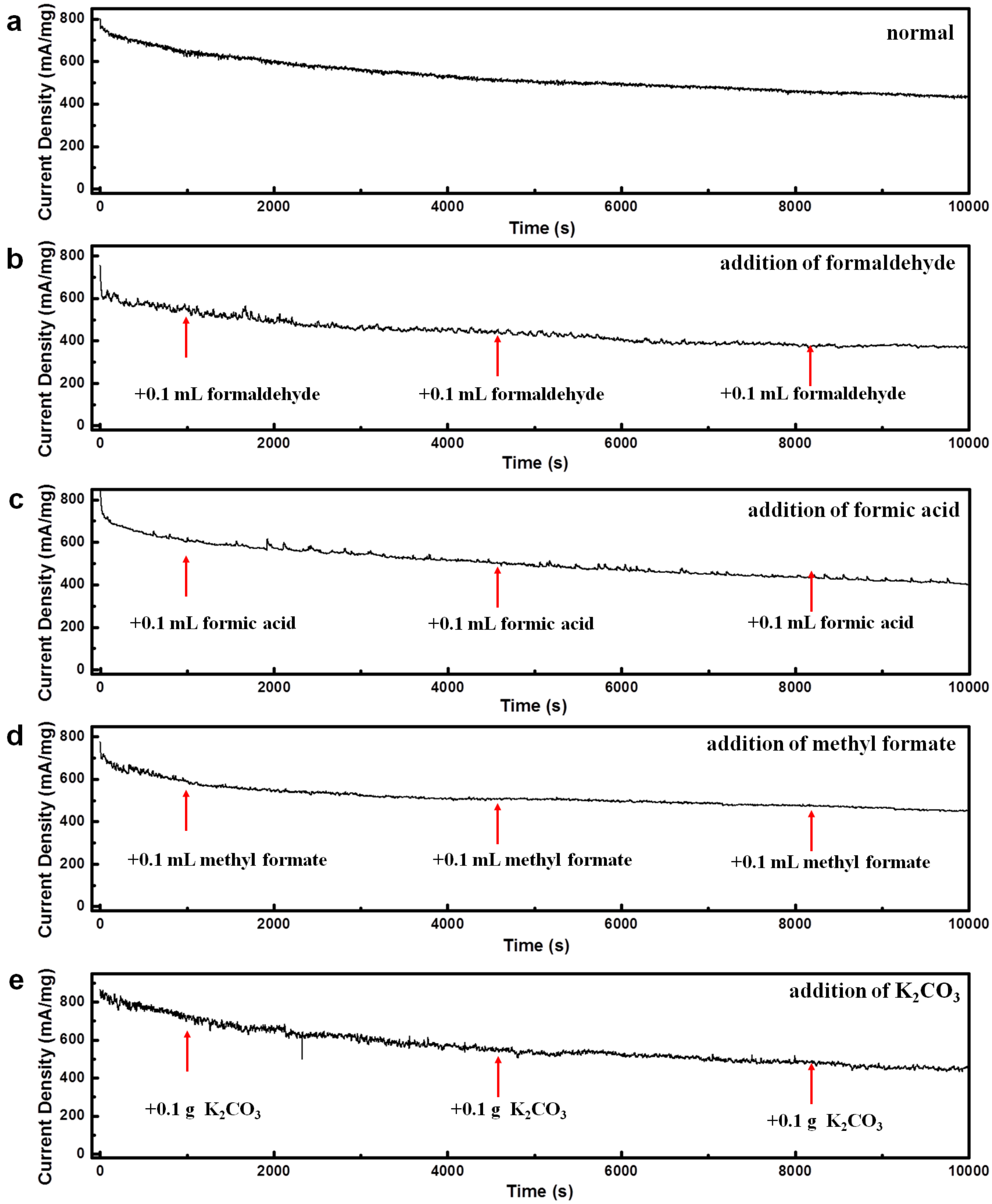

Supplementary Figure 15. (a) Typical chronoamperometric ($i \sim t$) curve of Pt/Ni(OH)$_2$/rGO-4 in 1 M methanol/1 M KOH, and its responses to the intentional additions of (b) formaldehyde, (c) formic acid, (d) methyl formate and (e) K_2CO_3 to the electrolyte. The data here reflect the high tolerance of our hybrid electrocatalysts to possible reaction intermediates and products.

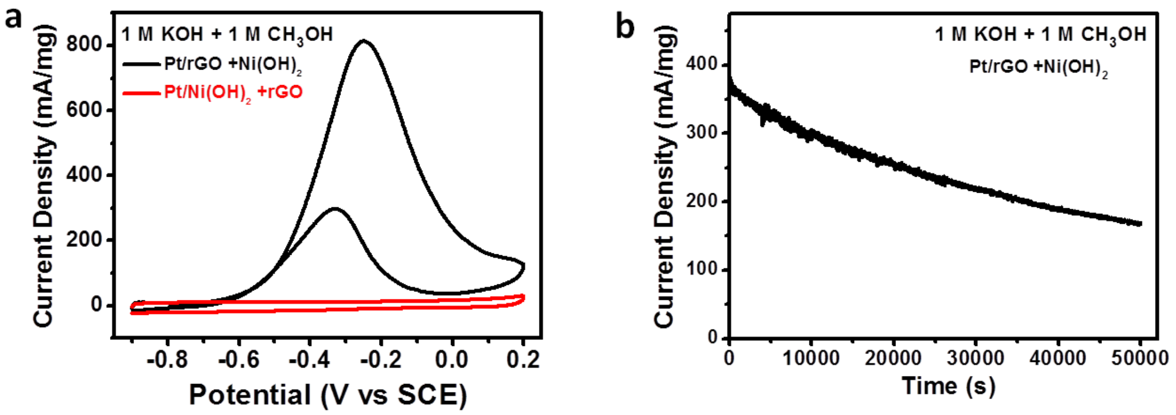

Supplementary Figure 16. (a) MOR CV curves of the physical mixtures of Pt/rGO and $Ni(OH)_2$ or Pt/$Ni(OH)_2$ and rGO. (b) Chronoamperometry of the physical mixture of Pt/rGO and $Ni(OH)_2$ at -0.3 V in 1 M methanol/1 M KOH solution. The poor MOR activity of the physical mixture of Pt/$Ni(OH)_2$ and rGO did not necessitate an evaluation of its durability.

Supplementary Table 1. Parameters used in the XANES and FT-EXAFS data work-up and fitting of [a]$Ni(OH)_2$/rGO-4, [b]Pt/$Ni(OH)_2$/gGO-4 and [c]Pt/rGO.

Absorption Edge	Background Subtraction		μ Spline Range / Å^{-1}	FT Range / Å^{-1}	Fitting Region / Å
	Pre-edge / eV	Post-edge / eV			
Ni K	8,132 − 8,250	8,380 − 9,176	1.08 − 14.77	[a]2.5 − 14.0<br>[b]2.5 − 12.0	[a]1.40 − 2.90 [b]1.40 − 3.10
Pt L_3	11,364 − 11,450	11,590 − 12,310	1.05 − 14.01	[b]2.5 − 12.0<br>[c]2.5 − 11.0	1.35 − 3.10

Supplementary Table 2. Pt and Ni wt% in the ternary hybrids and Pt/rGO hybrid determined from ICP-AES, their initial ECSA and ECSA after 50,000 s MOR electrocatalysis.

	Pt wt%	Ni wt%	Initial ECSA (m^2 g^{-1})	ECSA after 50,000 s MOR electroatalysis (m^2 g^{-1})
**20 wt% Pt/C**	20.0	0	36.1	22.2
**Pt/rGO**	69.9	0	49.5	34.0
**Pt/$Ni(OH)_2$/rGO-1**	16.6	32.9	12.5	6.0
**Pt/$Ni(OH)_2$/rGO-2**	32.2	22.1	23.0	18.7
**Pt/$Ni(OH)_2$/rGO-3**	37.2	15.9	51.4	37.2
**Pt/$Ni(OH)_2$/rGO-4**	42.5	10.5	64.1	47.1
**Pt/$Ni(OH)_2$/rGO-5**	49.6	5.6	59.0	36.8

Supplementary Table 3. A summary of the performances of MOR electrocatalysts in alkaline electrolytes available in literature.

Electrocatalyst	Electrolyte	Peak current from CV curves	Durability	Reference
Pt/Ni(OH)$_2$/rGO ternary hybrids	1 M KOH + 1 M methanol	1070 mA mg^{-1} or 150 mA cm^{-2}	90 % activity retention after 3600 s; 40 % activity retention after 50000 s; but fully recoverable	This work
Pd-MoS$_2$	0.5 M KOH + 1 M methanol	440 mA mg^{-1}	N/A	1
CoPd/C	0.1 M KOH + 0.1 M methanol	21.8 mA cm^{-2}	Dropped to zero after 3500 s	2
Pt on graphene and vanadium carbonitride	1 M KOH + 0.5 M methanol	45 mA cm^{-2}	60 % activity retention after 5000 s	3
PdAg/Ti$_{0.5}$Cr$_{0.5}$N	0.1 M KOH + 1 M methanol	844 mA mg^{-1}$_{Pd}$	60 % activity retention after 3600 s	4
PtNi/C	1 M NaOH + 1 M methanol	1.2 A mg^{-1}	N/A	5
AuPt/MWCNTs	0.5 M KOH + 1 M methanol	1.6 mA (cm^{-2} mg^{-1})	55 % activity retention after 3600 s	6
Pt/karst-Ni thin film	1 M KOH + 1 M methanol	14 mA cm^{-2}	30 % activity retention after 3600 s	7
Pt-poly(5-nitro indoled)	1 M KOH + 1 M methanol	25 mA cm^{-2}	10 % activity retention after 3600 s	8
Pd film	0.1 M NaOH + 0.5 M methanol	3 mA cm^{-2}	N/A	9
PtAu/RGO/GC	1 M KOH + 1 M methanol	1600 mA mg^{-1}$_{Pt+Au}$	20 % activity retention after 4000 s	10
Popcorn-like PtAu	1 M KOH + 1 M methanol	30 mA cm^{-2}	20 % activity retention after 6000 s	11
Pt/carbon sphere	1 M NaOH + 1 M methanol	48 mA cm^{-2}	10 % activity retention after 1200 s	12
PtNi alloy	0.5 M KOH + 2 M methanol	20 mA cm^{-2}	35 % activity retention after 3600 s	13
Pt-rich shell coated Ni nanoparticles	1 M KOH + 1 M methanol	300 mA mg^{-1}$_{Pt}$	N/A	14
Pt$_m$Ag	0.5 M KOH + 2 M methanol	3 mA ug^{-1}	75 % activity retention after 3600 s	15
Pd-NiO/C	1 M KOH + 1 M methanol	70 mA cm^{-2}	40 % activity retention after 3.8 h	16
PdAu/C	1 M KOH + 1 M methanol	900 mA mg^{-1}	45 % activity retention after 1000 s	17
Pt nanoflouers-TiO$_2$ nanotube arrays	0.5 M KOH + 1 M methanol	4 mA cm^{-2}	37.5 % activity retention after 3000 s	18

Pt-TiO_2/ITO	1 M KOH + 1 M methanol	1.8 mA cm^{-2}	40 % activity retention after 400 s	19

Supplementary Methods

Material preparation

Synthesis of graphene oxide (GO). GO was made by a modified Hummers' method using a lower concentration of oxidizing agent. First, 1 g of graphite flakes was grounded with 40 g of NaCl for 30 min to fine powders. The mixtures were then transferred to a vacuum filtration apparatus, and washed with copious amounts of water to remove NaCl salts. Remaining graphite powders were dried in an oven at 80 °C for over 30 minutes. Afterwards, powders were transferred to a 250 ml round bottom flask, and carefully added with 23 ml of concentrated sulfuric acid. The suspension was continuously stirred at room temperature for 24 h. Subsequently, the flask was heated in an oil bath at 40°C. 100 mg of $NaNO_3$ was added to the suspension and allowed to dissolve in 5 minutes, followed by the slow addition of 500 mg of $KMnO_4$. The solution was allowed to stir for 30 minutes. At the end of the reaction, a total of ~200 ml of water was slowly added to the flask over ~ 30 min while solution temperature is maintained below 45 ^{o}C. Extra $KMnO_4$ was decomposed by adding 10 ml of 30% H_2O_2. Reaction solution was then repeatedly centrifuged, washed with 5% HCl solution twice and then with copious amounts of water till pH > 4. Final precipitates were re-dispersed in 100 ml of water and bath sonicated for 30 min to completely exfoliate GO nanosheets. At last, the GO solution was subjected to centrifugation at 5000 rpm for 5 min. The crushed out solids were discarded, and the brown homogenous supernatant was collected for further use.

Synthesis of Pt/Ni(OH)$_2$/rGO ternary hybrids. The synthesis is typified by the preparation of Pt/Ni(OH)$_2$/rGO-4 as detailed below. GO nanosheets were collected from their aqueous solution by centrifugation at 15000 rpm for 1 h and re-dispersed in N, N-dimethyl formamide (DMF) to form a solution of 0.75 mg ml^{-1}. To 8 ml of this solution were then added 0.313ml of 0.2 M $NiAc_2$ aqueous

solution and 0.2 ml of concentrated ammonia solution (25 wt%). It was heated in an oil bath at 85 ^{o}C with magnetic stirring for 12 h. Reaction product from this step was collected and is denoted as $Ni(OH)_2$/GO-4, in which GO nanosheets are barely reduced at such mild temperatures. Subsequently, $Ni(OH)_4$/GO-4 was re-dispersed in 6 ml of ethylene glycol (EG) and 6 ml of water, added with 1.2 ml of 77 mM H_2PtCl_6/EG solution and 0.24 ml of sodium salt of poly(methacrylic acid) (PMAA) aqueous solution (30 wt%), and subjected to microwave heating (Galanz D8023CSL-K4) at 800 W for 90 s. The resulting product was collected by centrifugation, washed with water, and finally lyophilized. In the second step, GO nanosheets were thermally reduced so we use rGO to represent reduced graphene oxide in the final Pt/$Ni(OH)_2$/rGO-4 hybrids.

The preparation of other ternary hybrids follows the same procedure, except that different amounts of $NiAc_2$ and H_2PtCl_6 were added during the two-step reaction. In addition, we also prepared $Ni(OH)_2$/rGO by only skipping the Pt precursor in the second step under otherwise identical conditions (this has to be differentiated from $Ni(OH)_2$/GO as explained above). Pt/rGO was prepared by directly microwave heating of H_2PtCl_6 and GO in the presence of PMAA. rGO was also prepared by directly microwave heating of GO nanosheets in the presence of PMAA. Hybrids with less defective $Ni(OH)_2$ was prepared by collecting $Ni(OH)_2$/GO-4 hybrids obtained from the first-step reaction, and subjected them to an additional hydrothermal reaction at 180°C overnight prior to Pt growth (denoted as Pt/ht-$Ni(OH)_2$/rGO-4).

XAS studies

XANES and STXM. The spatially-resolved XANES at the C K-edge, O K-edge and Ni L-edge were obtained on the scanning transmission X-ray microscope (STXM) at the 10ID-1 Spectro-Microscopic (SM) beamline of the Canadian Light Source (CLS), a 2.9 GeV third

generation synchrotron source. In STXM, the monochromatic X-ray beam is focused by a Fresnel zone plate to a ~30 nm spot on the sample, and the sample is raster-scanned with synchronized detection of transmitted X-rays to generate a sequence (stack) of images over a range of photon energies across an elemental edge. Image stacks were acquired at the same selected region where a well-defined single graphene/hybrid sheet was identified. Spatially-resolved XANES spectra were extracted from the graphene/hybrid sheet directly from the image stacks. Chemical mapping was conducted by fitting the image stacks with the reference spectra of the relevant chemical components. More details of the STXM experimental and data analysis procedures can be found elsewher.[20-21]

XANES and EXAFS data processing. XANES/FT-EXAFS data processing and fitting were performed using WinXAS software;[22] parameters used in the data work-up and fitting are presented in the Supplementary Table 1. A k-weighting of 2 was used for both the Ni K and Pt L_3 edges. Uncertainties in the structural parameters obtained from FT-EXAFS fitting were determined using a previously established method,[23] which emulates that used by the popular IFEFFIT package.[24] Pt-O/Pt-Pt and Ni-O/Ni-Ni scattering paths (generated using FEFF8.2)[25] were included, and were observed to closely fit the experimental data. A fixed S_0^2 value of 0.9 and a single edge energy shift (ΔE_0) value were used to obtain each fitting curve.

Electrochemical measurements

General experimental setup. CH Instruments 660E potentiostat was used for all experiments. To prepare the working electrode, 1 mg of electrocatalysts were blended with 6 μl of 5 wt% Nafion solution in 0.125 ml of water and 0.125 ml of ethanol, and formed a homogenous ink with the assistance of at least 30 min vigorous sonication. Then 5 μl of the catalyst ink was drop-cast onto a glassy carbon electrode (CH Instruments) of 3 mm in diameter to achieve a catalyst loading density

of 0.28 mg cm^{-2} for all measurements. A graphite rod was used as the counter electrode. All potentials were measured and reported against a saturated calomel reference electrode (SCE). Results were benchmarked against commercial 20 wt% Pt/C and 20 wt% PtRu/C purchased from FuelCellStore.

Pre-activation. Prior to any electrochemical measurements, electrocatalysts were activated in N_2 saturated 1 M KOH by CV cycling in the potential range of -1.0~0.2 V at 200 mV s^{-1} for about 50 cycles until the curve stabilizes. Afterwards, their CV curves were carefully collected at a slow scan rate of 20 mV s^{-1}. From their integrated hydrogen desorption charge in the positive-going potential scan (about -0.9~-0.6 V), the electrochemical surface areas (ECSA) of different electrocatalysts were calculated according to the following equation:

$$\mathrm{ECSA} = \frac{\mathrm{Q_H}}{0.21\mathrm{mC\ cm^{-2}} \times \mathrm{m_{Pt}}} \qquad (1)$$

where Q_H is the hydrogen desorption charge, 0.21 mC cm^{-2} is the charge density associated with the deposition of a hydrogen monolayer on planar polycrystalline Pt, and m_{Pt} is the Pt mass in the working electrode from ICP-AES measurements.

MOR electrocatalysis. CV measurements were performed in 1 M methanol/1 M KOH in the potential range of -0.9~0.2 V at 20 mV s^{-1}. Durability of electrocatalysts was assessed by chronoamperometry at a constant potential of -0.3 V and with gentle stirring of electrolyte to minimize methanol concentration polarization. For the flow-cell experiments, we customized an electrochemical cell (~10 ml) with a liquid inlet and outlet. Electrolyte was forced to circulate at a rate of 6~7 ml/min by a micropump during the durability measurements. For the reactivation during long-term durability measurements, the working electrode after every 50,000 s experiment was switched to plain 1 M KOH electrode, and subjected to CV cycling in the potential range of -1.0~0.2

V at 200 mV s^{-1} for about 20 cycles. Reactivated working electrode was then switched back to fresh 1 M methanol/1 M KOH electrolyte for further evaluations.

CO stripping and CO poisoning experiments. For CO stripping measurements, a monolayer of CO was adsorbed on electrocatalysts by flowing a 10% CO/N_2 in 1 M KOH for 30 min while the working electrode was held at -0.96 V. Non-adsorbed CO was then removed by bubbling the electrolyte with N_2 for 15 min. Stripping measurements were initiated from -0.96 V and first scanned in the anodic direction at 10 mV s^{-1} for at least two consecutive cycles. The response of different electrocatalysts toward intentional CO poisoning in 1 M methanol/1 M KOH was recorded by bubbling 10% CO/N_2 to the electrolyte at the middle of chronoamperometric measurements at a constant potential of -0.3 V.

Supplementary References:

1 Yuwen, L. *et al.* General synthesis of noble metal (Au, Ag, Pd, Pt) nanocrystal modified MoS_2 nanosheets and the enhanced catalytic activity of Pd-MoS_2 for methanol oxidation. *Nanoscale* **6**, 5762-5769 (2014).

2 Xi, P. *et al.* Facile synthesis of Pd-based bimetallic nanocrystals and their application as catalysts for methanol oxidation reaction. *Nanoscale* **5**, 6124-6130 (2013).

3 Huang, T. *et al.* A high-performance catalyst support for methanol oxidation with graphene and vanadium carbonitride. *Nanoscale* **7**, 1301-1307 (2015).

4 Cui, Z., Yang, M. & DiSalvo, F. J. Mesoporous $Ti_{0.5}Cr_{0.5}N$ supported PdAg nanoalloy as highly active and stable catalysts for the electro-oxidation of formic acid and methanol. *ACS Nano* **8**, 6106-6113 (2014).

5 Jiang, Q., Jiang, L., Wang, S., Qi, J. & Sun, G. A highly active PtNi/C electrocatalyst for methanol electro-oxidation in alkaline media. *Catal. Commun.* **12**, 67-70 (2010).

6 Guo, X., Guo, D.-J., Qiu, X.-P., Chen, L.-Q. & Zhu, W.-T. A simple one-step preparation of high utilization AuPt nanoparticles supported on MWCNTs for methanol oxidation in alkaline medium. *Electrochem. Commun.* **10**, 1748-1751 (2008).

7 Chen, C.-S., Pan, F.-M. & Yu, H.-J. Electrocatalytic activity of Pt nanoparticles on a karst-like Ni thin film toward methanol oxidation in alkaline solutions. *Appl.Catal.B* **104**, 382-389 (2011).

8 Zhou, W., Zhai, C., Du, Y., Xu, J. & Yang, P. Electrochemical fabrication of novel platinum-poly(5-nitroindole) composite catalyst and its application for methanol oxidation in alkaline medium. *Int. J. Hydrogen Energy* **34**, 9316-9323 (2009).

9 Yang, Y. Y. *et al.* Infrared spectroelectrochemical study of dissociation and oxidation of methanol at a palladium electrode in alkaline solution. *Langmuir* **29**, 1709-1716 (2013).

10 Ren, F. *et al.* One-pot synthesis of a RGO-supported ultrafine ternary PtAuRu catalyst with high electrocatalytic activity towards methanol oxidation in alkaline medium. *J. Mater. Chem. A* **1**, 7255-7261 (2013).

11 Zheng, J.-N. *et al.* Popcorn-like PtAu nanoparticles supported on reduced graphene oxide: Facile synthesis and catalytic applications. *J. Mater. Chem. A* **2**, 8386 (2014).

12 Liu, Y., Yang, H., Li, X. & Mao, L. Pt nanoparticles supported on monodisperse carbon spheres for methanol oxidation in alkaline media. *Mater. Lett.* **106**, 287-289 (2013).

13 Xiong, L., Yang, X., Xu, M., Xu, Y. & Wu, D. Pt–Ni alloy nanoparticles supported on multiwalled carbon nanotubes for methanol oxidation in alkaline media. *J.Solid. State. Electr.* **17**, 805-810 (2012).

14 Fu, X.-Z., Liang, Y., Chen, S.-P., Lin, J.-D. & Liao, D.-W. Pt-rich shell coated Ni nanoparticles as catalysts for methanol electro-oxidation in alkaline media. *Catal. Commun.* **10**, 1893-1897 (2009).

15 Feng, Y.-Y., Bi, L.-X., Liu, Z.-H., Kong, D.-S. & Yu, Z.-Y. Significantly enhanced electrocatalytic activity for methanol electro-oxidation on Ag oxide-promoted PtAg/C catalysts in alkaline electrolyte. *J. Catal.* **290**, 18-25 (2012).

16 Ye, K.-H., Zhou, S.-A., Zhu, X.-C., Xu, C.-W. & Shen, P. K. Stability analysis of oxide (CeO_2, NiO, Co_3O_4 and Mn_3O_4) effect on Pd/C for methanol oxidation in alkaline medium. *Electrochim. Acta* **90**, 108-111 (2013).

17 Yin, Z. *et al.* Supported bimetallic PdAu nanoparticles with superior electrocatalytic activity towards methanol oxidation. *J. Mater. Chem. A* **1**, 9157 (2013).

18 Zhai, C. *et al.* Visible-light-assisted electrocatalytic oxidation of methanol using reduced graphene oxide modified Pt nanoflowers-TiO_2 nanotube arrays. *ACS Appl. Mater. Interfaces* **6**, 17753-17761 (2014).

19 Zhang, H. *et al.* Enhanced electrocatalytic performance for methanol oxidation on Pt–TiO_2/ITO electrode under UV illumination. *Int. J. Hydrogen Energy* **35**, 13290-13297 (2010).

20 Zhou, J. G. *et al.* Nanoscale chemical imaging and spectroscopy of individual RuO_2 coated carbon nanotubes. *Chem. Commun.* **46**, 2778-2780 (2010).

21 Zhou, J. G. *et al.* Imaging nitrogen in individual carbon nanotubes. *J. Phys. Chem. Lett.* **1**, 1709-1713 (2010).

22 Ressler, T. WinXAS: a program for x-ray absorption spectroscopy data analysis under MS-Windows. *J. Synchrotron Rad.* **5**, 118-122 (1998).

23 Zhang, W. *et al.* In situ electrochemical XAFS studies on an iron fluoride high-capacity cathode material for rechargeable lithium batteries. *J. Phys. Chem. C* **117**, 11498-11505 (2013).

24 Ravel, B. & Newville, M. ATHENA, ARTEMIS, HEPHAESTUS: data analysis for X-ray absorption spectroscopy using IFEFFIT. *J. Synchrotron Rad.* **2005** (2005).

25 Ankudinov, A. L., Ravel, B., Rehr, J. J. & Conradson, S. D. Real-space multiple-scattering calculation and interpretation of x-ray-absorption near-edge structure. *Phys. Rev. B* **58**, 7565--7576 (1998).
